# Distinct binding conformations of epinephrine with α- and β-adrenergic receptors

**DOI:** 10.1038/s12276-024-01296-x

**Published:** 2024-09-02

**Authors:** Jian-Shu Lou, Minfei Su, Jinan Wang, Hung Nguyen Do, Yinglong Miao, Xin-Yun Huang

**Affiliations:** 1 Department of Physiology and Biophysics, Weill Cornell Medical College of Cornell University, New York, NY 10065 USA; 2 Center for Computational Biology and Department of Molecular Biosciences, University of Kansas, Lawrence, KS 66047 USA; 3 Present Address: School of Pharmacy, Hangzhou Normal University, Hangzhou, Zhejiang 311121 China

**Keywords:** Chemokines, Drug discovery, Molecular biology, Cardiovascular biology

## Abstract

Agonists targeting α_2_-adrenergic receptors (ARs) are used to treat diverse conditions, including hypertension, attention-deficit/hyperactivity disorder, pain, panic disorders, opioid and alcohol withdrawal symptoms, and cigarette cravings. These receptors transduce signals through heterotrimeric Gi proteins. Here, we elucidated cryo-EM structures that depict α_2A_-AR in complex with Gi proteins, along with the endogenous agonist epinephrine or the synthetic agonist dexmedetomidine. Molecular dynamics simulations and functional studies reinforce the results of the structural revelations. Our investigation revealed that epinephrine exhibits different conformations when engaging with α-ARs and β-ARs. Furthermore, α_2A_-AR and β_1_-AR (primarily coupled to Gs, with secondary associations to Gi) were compared and found to exhibit different interactions with Gi proteins. Notably, the stability of the epinephrine–α_2A_-AR–Gi complex is greater than that of the dexmedetomidine–α_2A_-AR–Gi complex. These findings substantiate and improve our knowledge on the intricate signaling mechanisms orchestrated by ARs and concurrently shed light on the regulation of α-ARs and β-ARs by epinephrine.

## α vs. β-ARs: epinephrine signaling mechanisms revealed

Our bodies have a system, the sympathetic nervous system, that uses certain chemicals to control heart rate, blood pressure, etc. These chemicals, epinephrine and norepinephrine, work by activating proteins known as adrenergic receptors. Understanding these receptors could help treat diseases like high blood pressure and ADHD. This study used a method called cryo-electron microscopy to see how epinephrine interacts with these receptors. It compared how epinephrine and a similar drug, dexmedetomidine, interact with the receptors. The study found that epinephrine binds to the α and β types of the receptors differently, which could explain their different effects. This helps us understand how drugs that mimic or block epinephrine can treat diseases. This could lead to new, more effective drugs. Future research may use these findings to design better treatments for heart diseases and other conditions.

This summary was initially drafted using artificial intelligence, then revised and fact-checked by the author.

## Introduction

In the sympathetic nervous system, endogenous epinephrine and norepinephrine regulate various physiological functions by activating adrenergic receptors (ARs)^1^. There are nine distinct AR subtypes in humans: α_1A_, α_1B_, α_1D_, α_2A_, α_2B_, α_2C_, β_1_, β_2_, and β_3_^2^. Three distinct genes that encode α_2_-AR subtypes have been identified^3^. Mouse genetic studies with individual α_2_-AR gene deletions have shown distinct and overlapping physiological functions. While α_2A_-ARs decrease sympathetic outflow and blood pressure, α_2B_-ARs increase blood pressure^3^. Both α_2A_-AR and α_2C_-AR are needed to regulate neurotransmitter release^3^. α_2_-AR agonists are used to treat hypertension, attention-deficit/hyperactivity disorder, and other diseases^4–6^. Dexmedetomidine is a highly selective α_2_-AR agonist used for prolonged sedation in hospitalized patients, procedural sedation and general anesthesia and treating emergence delirium^7–9^. The pharmacological blockade of α_2A_-AR by a clinically used antagonist reduces Alzheimer’s disease-related pathology and improves cognitive deficits in an Alzheimer’s disease transgenic model^10^. All ARs belong to the G-protein-coupled receptor (GPCR) superfamily^11^. α_2_-ARs are Gi-coupled GPCRs that can inhibit adenylyl cyclase and decrease intracellular cAMP levels.

Epinephrine is a hormone and is produced primarily by the chromaffin cells of adrenal glands^12^. The release of epinephrine is triggered by stress and plays major roles in fight-or-flight responses by increasing blood flow to muscles, heart output, the pupil dilation response, and blood sugar levels by binding to α- and β-ARs^2,12^. Epinephrine activates the β_1_-AR–Gs signaling pathway to increase cardiac output, whereas it stimulates the α_2A_-AR–Gi signaling pathway to decrease blood pressure. Surprisingly, other than the epinephrine–α_1A_-AR–Gq structure we recently identified, little structural information is available for epinephrine bound to a GPCR–G-protein complex^13^. There are two structures of epinephrine bound to β_1_-AR or β_2_-AR alone with the nanobody 6B9, which stabilizes β-ARs in an active state^14,15^. Epinephrine binds β_1_-AR and β_2_-AR in an identical manner^14,15^.

GPCRs transduce signals through heterotrimeric G proteins. Although one GPCR is typically coupled to one family of G proteins (G_s_, G_i_, G_q_ or G_12/13_), a GPCR may activate more than one of the four families of G proteins. However, the selective coupling of GPCRs and G proteins is not well understood. While β_1_-AR is primarily coupled to Gs, it can also couple to Gi^16–20^. Gi-mediated β_1_-AR signaling is implicated in decreased receptor responsiveness in myocardial infarction^16–19^. On the other hand, α_2A_-AR is primarily coupled to Gi. Previously, we solved the cryo-EM structure of a β_1_-AR and Gi complex^21^. We examined how a single GPCR simultaneously transduces signals through two G proteins by a comparative structural analysis of β_1_-AR complexed with Gi or Gs^21^. A structural comparison of the α_2A_-AR–Gi and β_1_-AR–Gi complexes should increase our knowledge on the coupling selectivity of GPCR and G-protein.

Here, we used cryo-EM to determine the structures of α_2A_-AR signaling complexes with Gi and the endogenous agonist epinephrine or the synthetic agonist dexmedetomidine. These results provide structural information on the complexation of epinephrine with a GPCR and a G protein. We identified different conformations of epinephrine in complex with α_2A_-AR–Gi versus β-AR–6B9 nanobodies. Furthermore, we compared the structures of the α_2A_-AR–Gi complex and the β_1_-AR–Gi complex, revealing different coupling modes of Gi to α_2A_-AR and to β_1_-AR. Moreover, we performed Gaussian accelerated molecular dynamics (GaMD) simulations to investigate the activation of α_2A_-AR by agonists and activation of Gi by agonist-bound α_2A_-AR^22,23^. GaMD is an enhanced sampling method that works by adding a harmonic boost potential to reduce the system energy barriers^22,23^. Without the need to set predefined collective variables, GaMD provides unconstrained enhanced sampling and is advantageous for simulating complex biological systems, such as GPCR–G-protein complexes. The harmonic boost potential in GaMD exhibits a Gaussian distribution, which enables us to accurately recover the original biomolecular free energy landscapes by Gaussian approximation, i.e., cumulant expansion to the second order. Thus, through GaMD, unconstrained enhanced sampling and free energy calculations of biomolecules can be simultaneously achieved. Previously, GaMD has been successfully applied to explore the activation and agonist binding of GPCRs^24–26^ and was thus applied in the present study. We proposed a possible sequential order starting from the interaction between activated α_2A_-AR and the C-terminal half of the α5-helix in Gi and ending with the conformational modification of the GDP/GTP-binding pocket and the opening of the α-helical domain during Gi activation.

## Materials and methods

### Expression and purification of α_2A_-AR, Gα_i_, Gβ_1_ and Gγ_2_

An HA signal peptide and a Flag tag were fused to human α_2A_-AR, followed by the PreScission protease cleavage site, eGFP and an 8xHis tag at the C-terminus. The construct was expressed and purified from *Spodoptera frugiperda* Sf9 insect cells grown in ESF 921 protein-free medium (Expression Systems), as we previously described^21,27^. After infection for two days, approximately 2–3 million cells/ml were harvested and flash frozen at −80 °C until use. Thawed cell pellets were sonicated (15 s on/15 s off for 4 min) in buffer containing 25 mM Tris-HCl (pH 7.4), 1 mM EDTA (pH 8.0), 160 μg/ml benzamidine, 1 μg/ml leupeptin, 4 μg/ml aprotinin, 0.5 μM PMSF, 1 μg/ml pepstatin A, 50 μM dexmedetomidine or epinephrine. The membranes were precleared to remove intact cells and debris from the broken cells by centrifugation at 2000 rpm for 10 min. The supernatant was carefully transferred to ultracentrifuge tubes and spun at 40,000 rpm at 6 °C for 1 h. The supernatant was removed, and the pellets were scraped from the bottles. Receptors were extracted using a Dounce homogenizer with the following buffers: 25 mM Tris-HCl (pH 7.4), 0.5 M NaCl, 1% (wt/vol) n-dodecyl-b-d-maltopyranoside (DDM; Anatrace), 0.2% sodium cholate, 0.02% cholesteryl hemisuccinate (CHS), 160 μg/ml benzamidine, 1 μg/ml leupeptin, 4 μg/ml aprotinin, 0.5 μM PMSF, 1 μg/ml pepstatin A, 50 μM dexmedetomidine or epinephrine, and stirring for 1 h at 4 °C. The supernatant was then incubated with HisPur™ Ni-NTA Resin (Thermo Fisher Scientific) for 2 h. The resin was collected by centrifugation, washed four times with 25 mM Tris-HCl (pH 7.4), 0.5 M NaCl, 0.05% DDM, 50 μM dexmedetomidine or epinephrine, and then eluted with 25 mM Tris-HCl (pH 7.4), 0.5 M NaCl, 0.05% DDM, 250 mM imidazole, 50 μM dexmedetomidine or epinephrine. The eluted α_2A_-AR was concentrated and further purified by size-exclusion chromatography using a Superdex 200 Increase 10/300 column (GE Healthcare) preequilibrated with 20 mM HEPES (pH 7.0), 100 mM NaCl, 0.05% Lauryl Maltose Neopentyl Glycol (LMNG, Anatrace), 50 μM dexmedetomidine or epinephrine, and the peak fractions were pooled and concentrated for complex assembly.

N-terminally His-tagged full-length rat Gα_i_ dominant negative (G203A) proteins were expressed and purified from the *E. coli* strain BL21 (DE3), as we previously described^21,28^. The Gα_i1_(G203A) mutant was used to increase the formation of the α_2A_-AR–Gi protein complex. The cells were grown in 2 × YT medium at 37 °C until the OD600 reached 0.6. Protein expression was then induced by 100 μM IPTG and continued for 16 h at 16 °C. The cells were harvested by centrifugation, flash-frozen in liquid nitrogen and stored at −80 °C. For protein purification, cell pellets were thawed in lysis buffer containing 50 mM Tris (pH 8.0), 150 mM NaCl, 5 mM β-mercaptoethanol, 10% glycerol, 2 mM MgCl2, 1 mM EDTA, 0.1 mg/ml lysozyme, 10 μM GDP, 0.5 mM PMSF, 1 μg/ml leupeptin, 4 μg/ml aprotinin, and 1 μg/ml pepstatin A and further lysed by sonication (4 s on/4 s off for 2 min). Cell debris was removed by centrifugation at 20,000 × *g* for 40 min at 4 °C. The supernatant was then collected and incubated with HisPur™ Ni-NTA Resin (Thermo Fisher Scientific) with stirring for 2 h at 4 °C. The resin was then washed four times with lysis buffer containing 25 mM imidazole and eluted with lysis buffer containing 250 mM imidazole. Gαi was concentrated and further purified by size-exclusion chromatography using a Superdex 200 Increase 10/300 column preequilibrated with 50 mM Tris (pH 8.0), 150 mM NaCl, 5 mM β-mercaptoethanol, 10% glycerol, and 10 μM GDP. Purified Gai was concentrated, flash frozen in liquid nitrogen and stored at −80 °C.

Bovine Gβ_1_ and bovine His6-tagged soluble Gγ_2_ (C68S) were coexpressed and purified from Sf9 insect cells as we previously described^21,27^. Sf9 cells were coinfected with 25 ml of each baculovirus when the density of the insect cell culture reached 3 million cells/ml. Forty-eight hours post-infection, the cells were harvested by centrifugation, flash frozen in liquid nitrogen and stored at −80 °C. The cell pellets were thawed in 25 mM Tris (pH 7.5), 150 mM NaCl, 10% glycerol, 2 mM β-mercaptoethanol, 5 mM MgCl_2_, 10 μM GDP, 0.5 mM PMSF, 160 μg/ml benzamidine, 1 μg/ml leupeptin, 4 μg/ml aprotinin, and 1 μg/ml pepstatin A. The cells were lysed by sonication, and the cell debris was removed by centrifugation at 142,000 × *g* for 30 min. The supernatants were collected and incubated with HisPur™ Ni-NTA Resin (Thermo Fisher Scientific) with stirring for 2 h at 4 °C. The resin was then washed four times with lysis buffer containing 25 mM imidazole and eluted with lysis buffer containing 250 mM imidazole. The eluted protein was concentrated and further purified using a Superdex 200 Increase 10/300 column preequilibrated with 25 mM Tris (pH 7.5), 150 mM NaCl, and 2 mM β-mercaptoethanol. The purified Gβ_1_γ2 protein was concentrated, flash frozen in liquid nitrogen and stored at −80 °C.

### Protein complex assembly and purification

To assemble the α_2A_-AR–Gi complex, Gαi and Gβ_1_γ_2_ were mixed at a 1:1 molar ratio in the presence of 2 mM MgCl_2_. The mixture was incubated for 30 min and then mixed with α_2A_-AR at a 1.8:1 ratio in 500 μl of buffer containing 10 mM HEPES (pH 7), 100 mM NaCl, 0.1 mM TCEP, 0.02% LMNG, 2 mM MgCl_2_, 0.1 mM TCEP, 0.4 U of Apyrase (Sigma), and 1 mM dexmedetomidine or epinephrine. To remove the GFP tag on α_2A_-AR, PreScission protease was also added at a ratio of 1:10 (w:w). After an additional overnight incubation at 4 °C, the mixture was centrifuged at 16,000 × *g* for 10 min to remove any precipitants. The supernatant was then loaded onto a Superdex 200 Increase 10/300 column preequilibrated with 10 mM HEPES (pH 7), 100 mM NaCl, 0.1 mM TCEP, 0.02% LMNG and 25 µM dexmedetomidine or epinephrine. The elution fractions from a single peak containing the pure α_2A_-AR–Gi complex were concentrated to ~2 mg/ml and used directly for making cryo-EM grids.

### Cryo-EM data collection

Next, 3.5 µL of α_2A_-AR–Gi complex at a concentration of 2 mg/ml was applied to glow-discharged 400 mesh gold Quantifoil R1.2/1.3 holey carbon grids (Quantifoil Micro Tools) and vitrified using a Vitrobot Mark IV (Thermo Fisher Scientific/FEI) at 22 °C and 100% humidity. Micrographs were collected on a 300 kV Titan Krios electron microscope (Thermo Fisher Scientific/FEI) with a Gatan K3 direct electron detector (Gatan, Inc.) in superresolution mode at a nominal 81,000× magnification. For the epinephrine complex, 6454 movies in the defocus range of −0.8 to −1.8 μm were recorded with a total accumulated dose of 50 e^-^/Å^2^. For dexmedetomidine, 6766 movies in the defocus range of −0.8 to −2.0 μm were recorded with a total accumulated dose of 51 e^-^/Å^2^.

### Image processing, 3D reconstruction, modeling, and refinement

Superresolution movies were aligned, Fourier cropped twice, and dose-weighted using MotionCor2 implemented in Relion3.1-beta^29–31^. The effects of the contrast-transfer function were estimated with CTFFIND v4.1.8^32^. For the dexmedetomidine dataset, Relion Laplacian-of-Gaussian picking with minimum and maximum dimensions of 80 Å and 130 Å, respectively, was used to heavily overpick at a rate of approximately 1500 particles per micrograph. The resulting particle stacks of nearly 11 million particles were Fourier cropped twice and processed through 2 rounds of 2D classification in CryoSparc v2.15.0 to remove false-positives, receptors alone, or G-proteins alone^33^ (Supplementary Fig. 3). A stack of 639,275 intact complex particles was then subjected to 2 rounds of 3D classification in Relion. After 3D classification, a final stack of 188,480 high-resolution particles was selected and further polished by 2 rounds of Bayesian polishing and one round of CTF refinement in Relion. Nonuniform refinement of the polished particles in CryoSparc yielded a map with 3.2 Å resolution using the 0.143 Fourier shell correlation criterion. These consensus stacks were also subjected to G protein-focused refinement in CryoSparc. The G-protein focused refinement map showed significant improvement in the G-protein region compared to the consensus map. A model was built starting from X-ray crystal structures of the antagonist-bound α_2A_-AR (PDB code 6KUX) and Gi heterotrimer (PDB code 1GG2) in Coot v0.9.1, which, combined with consensus and G-protein focused refinement maps, were used to generate a composite map in Phenix v1.18.2^34,35^. The resulting maps were super-sampled in Coot to 0.8656 Å per pixel with a 320-voxel box. The α_2A_-AR-Gi model was real-space refined against the composite map in Phenix, and a work/free half-map pair was used to prevent overfitting (Supplementary Fig. 3). For the epinephrine dataset, the processing of the cryo-EM data was similar to that for the dexmedetomidine dataset, except that Relion 2D template-based autopicking was utilized to pick particles instead of Laplacian-of-Gaussian picking (Supplementary Fig. 2), resulting in 7,720,235 particles. The particle stacks were also Fourier cropped twice and processed through 2 rounds of 2D classification in CryoSparc v2.15.0 to remove false-positives, receptors alone, or G-proteins alone (Supplementary Fig. 2). A stack of 1,618,395 intact complex particles was then subjected to 3 rounds of 3D classification in Relion. A final stack of 713,558 high-resolution particles was selected after 3D classification and further polished by 1 round of Bayesian polishing in Relion. Nonuniform refinement of polished final particle stacks in CryoSparc resulted in a map with 2.8 Å resolution using the 0.143 Fourier shell correlation criterion. Since focused refinements of the epinephrine dataset did not further improve map quality, the consensus map was super-sampled in Coot to 0.8560 Å per pixel with 320 voxel boxes and used for model building and refinement. The binding poses of dexmedetomidine and epinephrine were initially determined by densities as well as their geometric restraints and were further supported by MD simulations^36^. All model statistics were validated using MolProbity^37^.

### Gaussian accelerated molecular dynamics (GaMD)

GaMD is an enhanced sampling method that works by adding a harmonic boost potential to reduce the system energy barriers^22,23^. When the system potential $V(\mathop{r}\limits^{ \rightharpoonup })$ is lower than the reference energy E, the modified potential ${V}^{* }(\mathop{r}\limits^{ \rightharpoonup })$ of the system is calculated as follows:

$${V}^{* }\left(\mathop{r}\limits^{ \rightharpoonup }\right)=V\left(\mathop{r}\limits^{ \rightharpoonup }\right)+\Delta V\left(\mathop{r}\limits^{ \rightharpoonup }\right)$$

1 $$\Delta V\left(\mathop{r}\limits^{ \rightharpoonup }\right)=\left\{\begin{array}{ll}\frac{1}{2}k{\left(E-V\left(\mathop{r}\limits^{ \rightharpoonup }\right)\right)}^{2},\,&V\left(\mathop{r}\limits^{ \rightharpoonup }\right) < E\\ 0,&{V}\left(\mathop{r}\limits^{ \rightharpoonup }\right)\ge E,\end{array}\right.$$

where *k* is the harmonic force constant. The two adjustable parameters E and k are automatically determined on three enhanced sampling principles. First, for any two arbitrary potential values ${v}_{1}(\mathop{r}\limits^{ \rightharpoonup })$ and ${v}_{2}(\mathop{r}\limits^{ \rightharpoonup })$ found on the original energy surface, if ${V}_{1}(\mathop{r}\limits^{ \rightharpoonup })\, <\, {V}_{2}(\mathop{r}\limits^{ \rightharpoonup })$, $\Delta V$ should be a monotonic function that does not change the relative order of the biased potential values; i.e., ${V}_{1}^{* }(\mathop{r}\limits^{ \rightharpoonup })\, <\, {V}_{2}^{* }(\mathop{r}\limits^{ \rightharpoonup })$. Second, if ${V}_{1}(\mathop{r}\limits^{ \rightharpoonup })\, <\, {V}_{2}(\mathop{r}\limits^{ \rightharpoonup })$, the potential difference observed on the smoothed energy surface should be smaller than that of the original surface, i.e., ${V}_{2}^{* }(\mathop{r}\limits^{ \rightharpoonup }){\,-\,V}_{1}^{* }(\mathop{r}\limits^{ \rightharpoonup })\, <\, {V}_{2}(\mathop{r}\limits^{ \rightharpoonup }){\,-\,V}_{1}(\mathop{r}\limits^{ \rightharpoonup })$. By combining the first two criteria and using the formulas ${V}^{* }(\mathop{r}\limits^{ \rightharpoonup })$ and $\Delta V$, we obtain

2 $${V}_{\max }\le E\le {V}_{\min }+\frac{1}{k},$$

where ${V}_{\min }$ and ${V}_{\max }$ are the system minimum and maximum potential energies, respectively. To ensure that Eq. 2 is valid, *k* must satisfy $k\le 1/\left({V}_{\max }-{V}_{\min }\right)$. Let us define $k={k}_{0}\cdot 1/\left({V}_{\max }-{V}_{\min }\right)$; then, $0{ < k}_{0}\le 1$. Third, the standard deviation (SD) of $\Delta V$ must be small enough (i.e., narrow distribution) to ensure accurate reweighting using cumulant expansion to the second order: ${\sigma }_{\Delta V}=k\left(E-{V}_{{avg}}\right){\sigma }_{V}\le {\sigma }_{0}$, where ${V}_{{avg}}$ and ${\sigma }_{V}$ are the average and SD of *ΔV* with *σ*_0_ as a user-specified upper limit (e.g., ${10k}_{B}T$) for accurate reweighting. When E is set to the lower bound $E={V}_{\max }$ according to Eq. 2, ${k}_{0}$ can be calculated as follows:

3 $${k}_{0}=\min \left(1.0,{k}_{0}^{{\prime} }\right)=\min \left(1.0,\frac{{\sigma }_{0}}{{\sigma }_{V}}\cdot \frac{{V}_{\max }-{V}_{\min }}{{V}_{\max }-{V}_{{avg}}}\right),$$

Alternatively, when the threshold energy E is set to its upper bound *E* = *V*_*min*_ + 1/*k*, *k*_0_ is set to:

4 $${k}_{0}={k}_{0}^{{\prime} {\prime} }\equiv \left(1-\frac{{\sigma }_{0}}{{\sigma }_{V}}\,\right)\cdot \frac{{V}_{\max }-{V}_{\min }}{{V}_{{avg}}-{V}_{\min }},$$

If ${k}_{0}^{{\prime} {\prime} }$ is calculated between 0 and 1. Otherwise, *k*_0_ is calculated using Eq. 3.

### System setup and simulation analysis

The structures of epinephrine–α_2A_-AR–G_i_ and dexmedetomidine–α_2A_-AR–G_i_ cryo-EM were used for setting up simulation systems to explore the stability of the complex. The missing residues in the ECL2 of α_2A_-AR (PLISEKKGGGGGPQPA, residue number 168–184) were modeled by SWISS-MODEL^38^. To explore the G-protein activation mechanism, another two models with the α-helical domain were built as follows: Gα_i_ in the dexmedetomidine–α_2A_-AR–G_i_ cryo-EM structure was substituted with the GDP-bound conformation of Gα_i_ (residues 11-354 of PDB 1GG2) with or without GDP. The missing residues in 1GG2 (349–354) were added. All four simulation systems were prepared with the CHARMM-GUI web server using a membrane protein input generator^39^. The receptor was inserted into a palmitoyl-oleoyl-phosphatidyl-choline (POPC) bilayer. All chain termini were capped with neutral patches (acetyl and methylamide). All the disulfide bonds in the complexes that were resolved in the cryo-EM structures were maintained in the simulations. The systems were solvated in 0.15 M NaCl solution at 310 K. The CHARMM36m parameter set was used for the receptor, lipids and GDP^40–42^. The CGenFF 2.4.0 parameters were used for epinephrine and dexmedetomidine^43^. Force field parameters with high penalties were optimized with FFParm^44^. GaMD simulations of these systems followed a protocol similar to that used in previous studies of GPCRs^24,26^. For each of the complex systems, initial energy minimization, thermalization, and 20 ns cMD equilibration were performed using NAMD2.12^45^. A cutoff distance of 12 Å was used for the van der Waals and short-range electrostatic interactions, and the long-range electrostatic interactions were computed with the particle‒mesh Ewald summation method^46^. A 2-fs integration time step was used for all MD simulations, and a multiple-time-stepping algorithm was used with bonded and short-range nonbonded interactions computed every time step and long-range electrostatic interactions every two timesteps. The SHAKE algorithm was applied to all hydrogen-containing bonds. The NAMD simulation started with equilibration of the lipid tails. With all other atoms fixed, the lipid tails were energy minimized for 1000 steps using the conjugate gradient algorithm and melted with a constant number, volume, and temperature (NVT) run for 0.5 ns at 310 K. The four systems were further equilibrated using a constant number, pressure, and temperature (NPT) run at 1 atm and 310 K for 10 ns with 5 kcal/(mol^.^ Å^2^) harmonic position restraints applied to the protein and ligand atoms. Final equilibration of each system was performed using an NPT run at 1 atm pressure and 310 K for 0.5 ns with all atoms unrestrained. After energy minimization and system equilibration were complete, conventional MD simulations were performed on each system for 20 ns at 1 atm pressure and 310 K with a constant ratio constraint applied on the lipid bilayer in the X-Y plane.

With the NAMD output structures, the system topology and CHARMM36m force field files, the *ParmEd* tool in the AMBER package was used to convert the simulation files into the AMBER format^47^. The GaMD module implemented in the GPU version of AMBER20 was then applied to perform the simulations^22,47^. GaMD simulations included an 8-ns short cMD simulation used to collect the potential statistics for calculating GaMD acceleration parameters and a 48-ns equilibration after the boost potential was added. Finally, three independent 500-ns GaMD simulations with randomized initial atomic velocities were performed for the epinephrine–α_2A_-AR–G_i_ and dexmedetomidine–α_2A_-AR–G_i_ complexes without the α-helical domain. More replicas and longer simulations are needed to capture the G protein activation process because this process is slow. Therefore, four independent 3000-ns GaMD production simulations with randomized initial atomic velocities were performed for the intact G-protein-bound dexmedetomidine–α_2A_ complex with or without GDP. The average and SD of the system potential energies were calculated every 800,000 steps (1.6 ns). All GaMD simulations were run at the “dual-boost” level by setting the reference energy to the lower bound. One boost potential was applied to the dihedral energetic term, and the other was applied to the total potential energetic term. The upper limit of the boost potential SD, σ_0,_ was set to 6.0 kcal/mol for the dihedral and total potential energetic terms. Similar temperature and pressure parameters were used as in the NAMD simulations.

CPPTRAJ was used to analyze the GaMD simulations^48^. Snapshots of all three GaMD production simulations (1500 ns in total) were combined for structural clustering to identify the representative agonist binding conformations. The hierarchical agglomerative algorithm in CPPTRAJ was applied for clustering. The cutoff was set to 2.0 Å for the RMSD of the agonist and surrounding receptor residues (residues within 4 Å of agonist) to form a cluster. Interactions between epinephrine and the receptor were measured using the distance between the CG atom of D128 and the N1/O3 atoms of epinephrine, the OH atom of Y431 and the N1 atom of epinephrine, the OG atom of S215 and the O1 atom of epinephrine, and the OG atom of S219 and the O2 atom of epinephrine. Interactions between dexmedetomidine and the receptor were measured using the distance between the O atom of F427 and the N2 atom of dexmedetomidine, OG in the side chain of Y431 and the N2 atom of dexmedetomidine, and the O atom from the backbone of Y431 and the N2 atom of dexmedetomidine. The distance between the NZ atoms of K46 in the P-loop and the CG atom of D200 in β3 of the Switch II region in Gα_i_ was used to measure the salt bridge formed between K46 and D200. The degree of domain separation (Ras-like domain and α-helical domain) was measured by the distance between residue E276 in the Ras-like domain and residue A138 in the α-helical domain. The C-terminal α5−helix RMSD was measured relative to the α5-helix RMSD in the cryo-EM structure of α_2A_-AR–Gi. The time courses of these reaction coordinates obtained from the GaMD simulation are plotted in Figs. 2, 3, 6, 7, and Supplementary Fig. 11.

### cAMP measurement

Gα-depleted HEK293 cells (in which the genes for all Gα subunits expressed in HEK cells were mutated by using the CRISPR/Cas system)^49^ were cotransfected with α_2A_-AR and Gα_i1_ or β_1_-AR and Gα_i1_ for the studies presented in Supplementary Fig. 9. CHO cells were transfected with wild-type or mutant α_2A_-AR plasmids for the studies presented in Supplementary Fig. 8. The cells were starved from serum overnight, washed twice with Hank’s balanced salt solution containing 25 mM HEPES-NaOH (pH 7.4) and 0.1% BSA, and incubated in buffer containing 0.5 mM IMBX (Sigma) for 20 min at room temperature^27^. For the Gi-mediated cAMP inhibition assay, the cells were first stimulated with 20 μM forskolin for 20 min at 37 °C. Dexmedetomidine was added to cells cotransfected with α_2A_-AR and Gα_i1_. Isoproterenol was used to treat cells cotransfected with β_1_-AR and Gα_i1_. To terminate the reaction, the medium was aspirated and the solution was immediately treated with 0.1 M HCl for 10 min at room temperature. The cells were harvested by centrifugation, and the supernatant was collected for determination of cAMP concentration in triplicate with a Direct Cyclic AMP Enzyme Immunoassay kit (Enzo Life Sciences). The cAMP assays were repeated three times, and the data are presented as the means ± SDs of three independent experiments. The analysis was performed using the log(agonist) vs. response function of Prism 9 (GraphPad)^27,50^.

### Quantification and statistical analysis

In Supplementary Figs. 8 and 9, the cAMP assays were repeated three times, and the data are presented as the means ± SDs of three independent experiments. The analysis was performed using the log(agonist) vs. response function of Prism 9 (GraphPad), as indicated in the figure legends. The cryo-EM data collection and refinement statistics are listed in Supplementary Table 1.

## Results

### Cryo-EM structures of the signaling complexes of epinephrine–α_2A_-AR–Gi and dexmedetomidine–α_2A_-AR–Gi

**Fig. 1:**
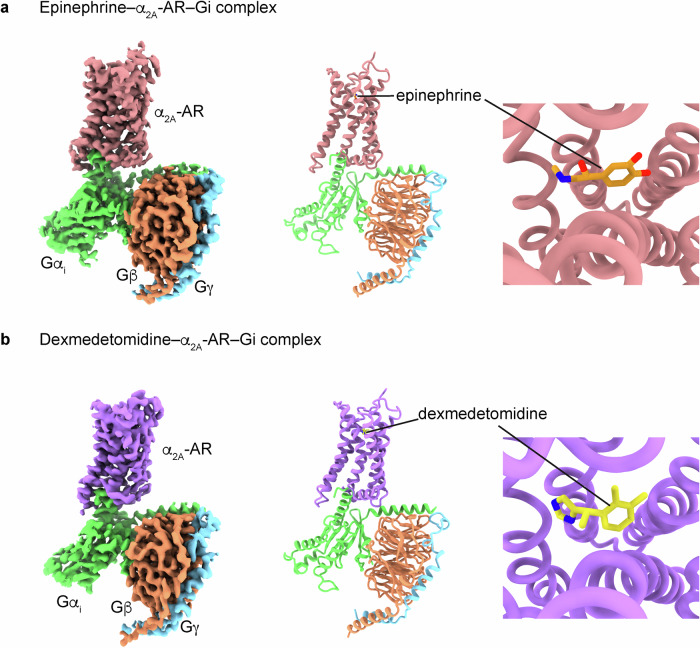
Cryo-EM structures of the complexes of epinephrine–α2A-AR–Gi and dexmedetomidine–α_2A_-AR–Gi. **a** The density map, model and ligand-binding pocket of epinephrine–α_2A_-AR–Gi are shown. **b** The density map, the model and the ligand-binding pocket of dexmedetomidine–α_2A_-AR–Gi are shown. Epinephrine-bound α_2A_-AR is colored in pink. Dexmedetomidine-bound α_2A_-AR is colored in purple. Gα_i1_ in green. Gβ in orange. Gγ in blue. Epinephrine and dexmedetomidine are colored in orange and yellow, respectively. Oxygen and nitrogen atoms are depicted in red and blue, respectively.

**Fig. 2:**
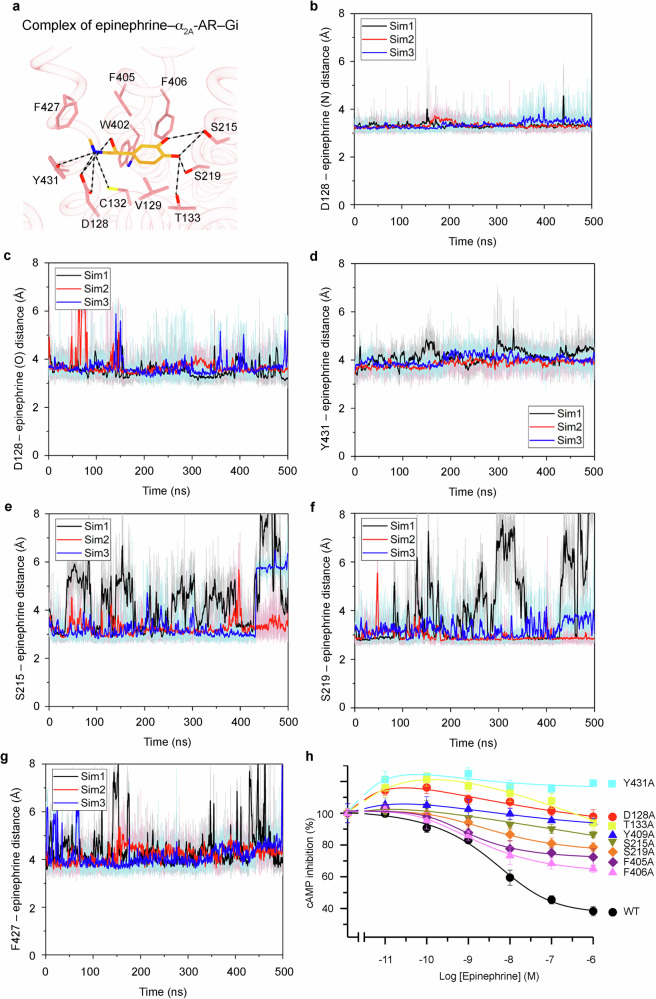
Interactions between epinephrine and α_2A_-AR. **a** Schematic diagram of the epinephrine-binding pocket of α_2A_-AR from the cryo-EM structure. Hydrogen bonds are depicted as dashed black lines. **b**–**f** Time courses of the interactions between epinephrine and specific residues of α_2A_-AR were calculated from the GaMD simulations: the distance between the CG atom of D128 and the N1 atom of epinephrine (**b**), the CG atom of D128 and the O3 atoms of epinephrine (**c**), the OH atom of Y431 and the N1 atom of epinephrine (**d**), the OG atom of S215 and the O1 atom of epinephrine (**e**), the OG atom of S219 and the O2 atom of epinephrine (**f**), and the phenyl group of F427 and the methyl group of epinephrine (**g**). **h** Functional studies of the epinephrine-interacting residues of α_2A_-AR. The effects of wild-type and mutant α_2A_-AR on the cAMP inhibition signaling initiated by epinephrine were studied. The data are shown as the means ± SDs of three independent experiments.

**Fig. 3:**
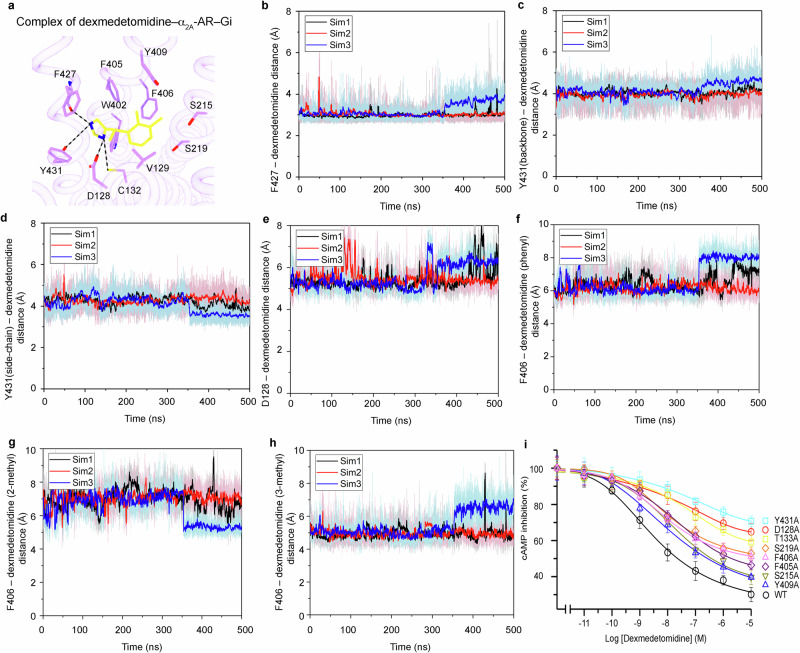
Interactions between dexmedetomidine and α_2A_-AR. **a** Schematic diagram of the dexmedetomidine binding pocket of α_2A_-AR from the cryo-EM structure. Hydrogen bonds are depicted as dashed black lines. **b**–**d** Time courses of the interactions between dexmedetomidine and specific residues of α_2A_-AR were calculated from the GaMD simulations: the distance between the O atom of F427 and the N2 atom of dexmedetomidine (**b**), OG in the side chain of Y431 and the N2 atom of dexmedetomidine (**c**), the O atom in the backbone of Y431 and the N2 atom of dexmedetomidine (**d**), the CZ atom of D128 and the nitrogen atom of dexmedetomidine (**e**), the phenyl group of F406 and the phenyl group of dexmedetomidine (**f**), the phenyl group of F406 and the 2-methyl group of dexmedetomidine (**g**), and the phenyl group of F406 and the 3-methyl group of dexmedetomidine (**h**). **i** Functional studies of the dexmedetomidine-interacting residues of α_2A_-AR. The effects of wild-type and mutant α_2A_-AR on the cAMP inhibition signaling initiated by dexmedetomidine were examined. The data are shown as the means ± SDs of three independent experiments.

To investigate the mechanism underlying the molecular recognition of epinephrine by an α-AR, we solved the structures of human α_2A_-AR bound to epinephrine (an endogenous agonist) or dexmedetomidine (a synthetic agonist) and in complex with its cognate signaling Gi heterotrimer (Gα_i_(G203A)Gβ_1_Gγ_2_). We used Gα_i1_(G203A) to increase the formation of the ligand–α_2A_-AR–Gi protein complex without using any stabilizing nanobodies (Supplementary Fig. 1)^51^. We solved a 2.8 Å cryo-EM structure of the epinephrine-bound α_2A_-AR and Gi signaling complex (Fig. 1a, Supplementary Fig. 2, and Supplementary Table 1) and a 3.2 Å cryo-EM structure of α_2A_-AR–Gi in complex with dexmedetomidine (Fig. 1b, Supplementary Fig. 3, and Supplementary Table 1). Overall, the structures of the α_2A_-AR–Gi complex are similar in the presence of epinephrine and dexmedetomidine, with a root-mean-square deviation (RMSD) of 1.1 Å over 865 Cα atoms (Fig. 1 and Supplementary Fig. 4). However, there are local conformational differences, especially in the ligand-binding pockets (Figs. 2, 3). While some of the interacting residues are common to both ligands, epinephrine and dexmedetomidine each form different interactions in the orthosteric ligand-binding pocket (Supplementary Figs. 5, 6 and 7).

### Distinct conformations of epinephrine binding to α_2A_-AR and β-ARs

Epinephrine, a chiral endogenous full agonist for all ARs, displays different binding conformations when interacting with α_2A_-AR and β-ARs (Fig. 2a and Supplementary Fig. 6). The unique conformations of epinephrine within these receptor complexes shed light on the structural and functional implications of these variations. In the structure of the epinephrine–α_2A_-AR–Gi complex, the β-carbon hydroxyl group is oriented toward the extracellular side (Fig. 2a and Supplementary Fig. 6). Notably, the N-methyl group is directed toward the transmembrane domain (TM) 7 residue F427^7.38^ (superscript denotes Ballesteros-Weinstein numbering)^52^ (Fig. 2a). Moreover, the meta-hydroxyl group of epinephrine interacts with S215^5.43^, and the para-hydroxyl group interacts with S215^5.43^, S219^5.46^, and T133^3.37^ (Fig. 2a). Additionally, the amino group interacts with D128^3.32^ and Y431^7.42^ (Fig. 2a), forming a comprehensive interaction network.

To validate the conformation of epinephrine within the cryo-EM structure of the epinephrine–α_2A_-AR–Gi complex, GaMD simulations were conducted. These simulations indicated that epinephrine forms consistent interactions with adjacent receptor residues, which is consistent with the cryo-EM structure. Although slight fluctuations were observed across multiple GaMD simulations (Fig. 2b–g), the overall conformation of epinephrine remained stable and aligned with that observed in the cryo-EM structure. Hydrogen bonds were formed between epinephrine and D128^3.32^, with average distances of 3.32 ± 0.15 Å and 3.63 ± 0.63 Å (Fig. 2b, c). Additionally, the hydrogen bonds formed between epinephrine and the receptor residues Y431^7.42^, S215^5.43^ and S219^5.46^ were measured at 4.20 ± 0.15 Å, 3.74 ± 0.88 Å and 3.45 ± 0.79 Å, respectively (Fig. 2d–f). A strong CH3-Π interaction between epinephrine and F427^7.38^ formed at a distance of 4.10 ± 0.65 Å (Fig. 2g). This reaffirms the reliability of the cryo-EM structure of the epinephrine–α_2A_-AR–Gi complex. To substantiate the insights garnered from cryo-EM and GaMD simulations, functional studies were carried out by mutating the residues that interacted with epinephrine on α_2A_-AR. The data extracted from these studies provide compelling support for the pivotal roles played by these residues in α_2A_-AR signaling (Fig. 2h and Supplementary Fig. 8).

Although structural data concerning epinephrine in complex with β-ARs and G-proteins remain scarce, we leveraged available information from epinephrine-bound β_1_-AR and β_2_-AR structures stabilized by nanobody 6B9 (PDB: 7BTS and 4LDO, respectively)^14,15^. The conformations of epinephrine within these β-AR complexes are identical. However, the conformation of epinephrine could differ in β-AR-G-protein complexes compared with β-AR-nanobody 6B9 complexes. The differing orientations of the β-carbon hydroxyl and N-methyl groups of epinephrine when complexed with α_2A_-AR and β-ARs stand out. Notably, this disparity arises due to the rotation around the chemical bond that links the catechol ring and β-carbon of epinephrine (Supplementary Fig. 6a). The critical hydrogen bond interactions of N363^7.38^ (in β_1_-AR) and N312^7.38^ (in β_2_-AR) with the β-carbon hydroxyl and N-methyl groups of epinephrine underscore their functional relevance^14,15^. Correspondingly, F427^7.38^ in α_2A_-AR occupies a similar position, participating in hydrophobic interactions with the N-methyl group of epinephrine (Fig. 2a). These structural findings align well with previous functional investigations, revealing the distinct roles of specific residues in subtype-specific ligand recognition^53,54^. Taken together, the multifaceted structural findings obtained by examining the interactions of epinephrine with α_2A_-AR and β-ARs enhance our knowledge of receptor‒ligand dynamics and functional outcomes. N312^7.38^ of β_2_-AR was shown to be essential for epinephrine binding and signaling^54^. Moreover, the N312^7.38^ mutation in F abolished the binding and signaling of β_2_-AR by epinephrine^54^. Furthermore, F427^7.38^ at the equivalent position was shown to be critical for α_2A_-AR function^53^. The F427^7.38^ mutation to N in α_2A_-AR increased the binding of β-AR antagonists while decreasing the binding of epinephrine to this mutant α_2A_-AR^53^. Researchers have functionally demonstrated that N (conserved in all β-ARs) or F (conserved in all α-ARs) at this position of TM7 are the main subtype determinant (α-ARs versus β-ARs)^55^. The structural studies of β-ARs and our current study of α_2A_-AR in complex with epinephrine provide the structural basis for subtype-specific ligand recognition. These insights further knowledge on the intricate ligand recognition mechanisms that are intrinsic to different AR subtypes.

### Interaction of dexmedetomidine and α_2_-ARs

Dexmedetomidine is a selective agonist for α_2_-AR^56–58^. The conformation of dexmedetomidine shows flexibility in the complexes of α_2A_-AR–Gi, α_2B_-AR–Gi–the antibody fragment scFv16 (which binds to the interface between Gα_i_ and Gβ and was used to stabilize the entire complex) (PDB: 6K42), and α_2A_-AR–Go–scFv16 (PDB: 7EJA)^59,60^(Supplementary Fig. 7). While the two methyl groups on the phenyl ring are in similar positions, the imidazole rings rotate and are in different positions in the different structural complexes (Supplementary Fig. 7). In the complex of dexmedetomidine–α_2A_-AR-Gi, the conserved and critical S215^5.43^ and S219^5.46^ residues (which interact with the hydroxyl groups on the catechol ring of epinephrine) are located near the two methyl groups on the phenyl ring of dexmedetomidine; however, the residues do not interact with these groups directly (Fig. 3a). Researchers have shown that S215^5.43^ and S219^5.46^, as well as their corresponding Ser residues in all other ARs, are critical for the agonist-induced activation of ARs^61^. Even though the imidazole group shows greater flexibility, the most interactions occur with F427^7.38^ and Y431^7.42^ (Fig. 3a). Furthermore, we employed GaMD simulations to verify the conformation of dexmedetomidine in the complex of dexmedetomidine–α_2A_-AR-Gi. Interactions between dexmedetomidine and residues F427^7.38^ and Y431^7.42^ in α_2A_-AR were similar in the GaMD simulations and in our cryo-EM structure (Fig. 3b–h). Hydrogen bonds were formed between dexmedetomidine and the receptor residues F427^7.38^ and Y431^7.42^ (which are both the backbone and the side chain), with average distances of 3.08 ± 0.25 Å, 4.06 ± 0.32 Å and 4.19 ± 0.37 Å, respectively (Fig. 3b–d). A salt-bridge interaction was observed between dexmedetomidine and D128^3.32^, with the CZ atom of D128 (negative charge center) and the N atom of dexmedetomidine (positive charge center) maintaining an average distance of 5.47 ± 0.45 Å (Fig. 3e). Hydrophobic interactions between dexmedetomidine and F427^7.38^ occurred, and the phenyl group of F427^7.38^ interacted with the phenyl group, 2-methyl group, and 3-methyl group of dexmedetomidine at distances of 6.43 ± 0.62 Å, 6.85 ± 0.66 Å, and 5.12 ± 0.50 Å, respectively (Fig. 3f–h). Moreover, we mutated the dexmedetomidine-interacting residues on α_2A_-AR, and functional studies supported their roles in dexmedetomidine-initiated α_2A_-AR signaling (Fig. 3i and Supplementary Fig. 8).

### Activation of α_2A_-AR by epinephrine and dexmedetomidine

**Fig. 4:**
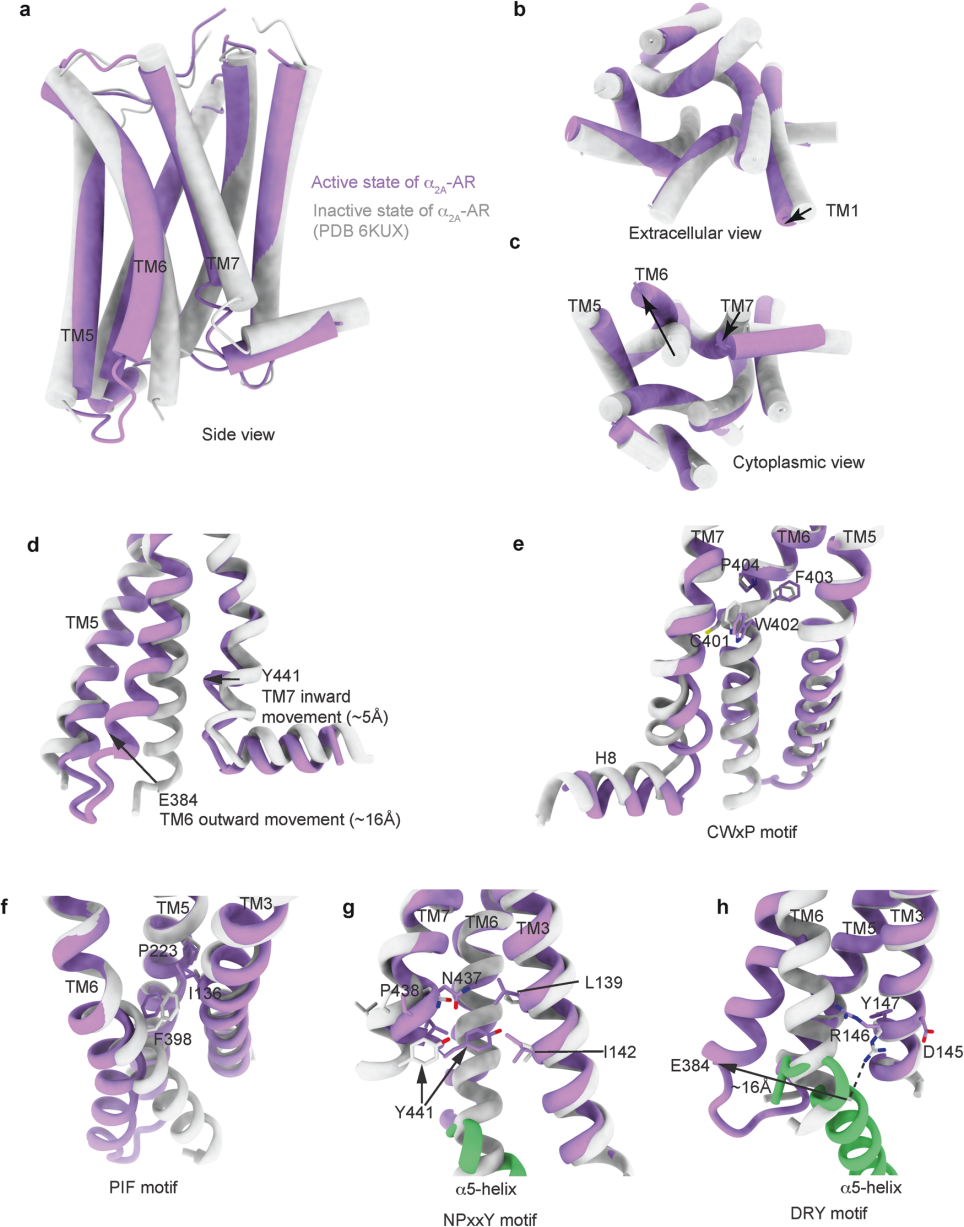
Activation of α_2A_-AR. **a**–**c** Differential images of the inactive state of α_2A_-AR (gray; PDB 6KUX) and the active state of α_2A_-AR in complex with Gi (this work). **d** The movements of TM6 and TM7 during α_2A_-AR activation are shown. **e** Conformational changes in the CWxP motif during α_2A_-AR activation are shown. **f** Conformational changes in the PIF motif during α_2A_-AR activation are shown. **g** Y441 within the NPxxY motif packs against L139 and I142 in the active state of α_2A_-AR. **h** The ionic lock between R146 (within the DRY motif) and E384 located at the cytoplasmic end of TM6 is disrupted in the active state of α_2A_-AR. The ionic bonding between R146 and E384 is indicated by a dashed black line.

We investigated the mechanism by which α_2A_-ARs are activated by epinephrine and dexmedetomidine. We compared the structures of α_2A_-AR in its inactive and active states. The inactive state of α_2A_-AR was captured by binding with the antagonist RS 79948 (PDB: 6KUX) or the partial agonist RES (PDB: 6KUY)^62^. Compared with these inactive state X-ray crystal structures, the active state α_2A_-AR in the complexes of epinephrine–α_2A_-AR–Gi and dexmedetomidine–α_2A_-AR–Gi shows characteristic conformational changes for class A GPCR activation (Fig. 4a).

The overall root-mean-square deviation between the structures of α_2A_-AR in the active and inactive states is 2.7 Å for 257 Cα atoms. The largest structural changes upon activation occurred on the cytoplasmic side of α_2A_-AR (Fig. 4b, c), with an outward rotation of TM6 by ~16 Å (measured at the Cα of E384^6.30^) and an inward ~5 Å movement of TM7 (measured at the Cα of Y441^7.53^) (Fig. 4d). In addition to these TM conformational changes, the side chains of certain residues are rearranged as part of the α_2A_-AR activation process. Immediately below the orthosteric ligand pocket, the rotameric change in W402^6.48^ (within the CWxP motif) indicates that TM6 in class A GPCRs opens for G-protein engagement^63^ (Fig. 4e). The layer below the CWxP motif is the PIF motif (P223^5.50^, I136^3.40^, and F398^6.44^) (Fig. 4f). Closer to the G protein-interacting site below the PIF motif, the highly conserved NPxxY motif at the cytoplasmic end of TM7 is another key microswitch of GPCR activation^63^ (Fig. 4g). TM7 rotates around the NPxxY motif. This shifts Y441^7.53^ toward the position occupied by TM6 in the inactive structure (Fig. 4g). Among the G protein-interacting residues, the rearrangement of side chains in the highly conserved D(E)/RY motif in TM3 is critical for GPCR activation^63^ (Fig. 4h). In inactive α_2A_-AR, the ionic-lock salt bridge is preserved between the side chains of R146^3.50^ and D145^3.49^, but it is broken in the active state α_2A_-AR structure (Fig. 4h). Additionally, R146^3.50^ forms a salt bridge with E384^6.30^ in the inactive state of α_2A_-AR, but this interaction is disrupted in the active state of α_2A_-AR (Fig. 4h). In the active state structure, the C-terminal end of the α5-helix of Gα_i_ occupies the space originally occupied by E384^6.30^ in the inactive state (Fig. 4h). The new position of E384^6.30^ in the active state is ~16 Å outward (Fig. 4h). The R146^3.50^ side chain forms a new packing interaction with C351 in the α5-helix of Gα_i_ (Fig. 4h). Therefore, α_2A_-ARs undergo conformational changes that propagate from the orthosteric ligand-binding site to the Gi-interacting site during activation. Similar conformational changes are observed in the epinephrine–α_2A_-AR–Gi and dexmedetomidine–α_2A_-AR–Gi structures.

### Molecular recognition of Gi by α_2A_-AR and β_1_-AR

**Fig. 5:**
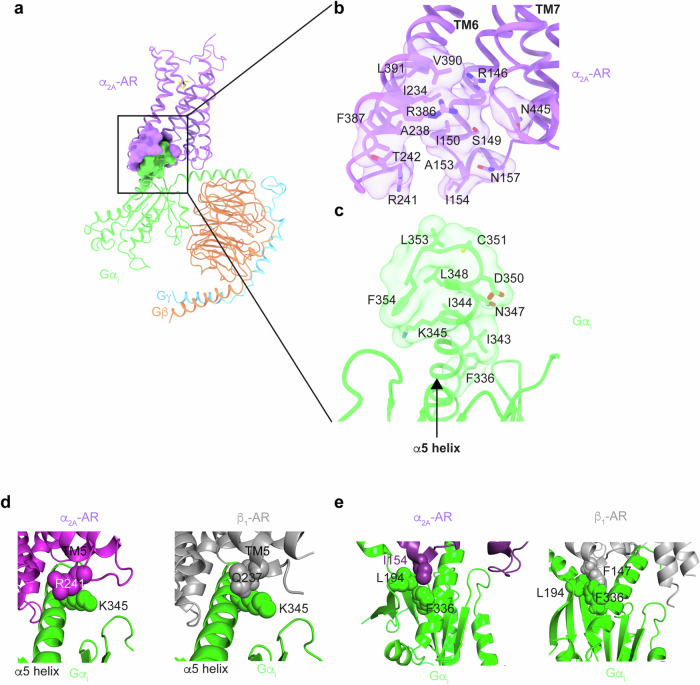
Interactions between Gi and α_2A_-AR or β_1_-AR. **a**–**c** Details of the interactions between Gi and α_2A_-AR. **d** K345 from Gi interacts with both α_2A_-AR and β_1_-AR. **e** Different residues in ICL2 (I154 from α_2A_-AR or F147 from β_1_-AR) contribute to the hydrophobic interactions with L194 from the β_2_-β_3_ loop and F336 from the C-terminal α5-helix of Gα_i_.

To determine the molecular mechanism by which α_2A_-AR activates Gi, we investigated how α_2A_-AR recognizes Gi during the activation process. As revealed by the cryo-EM structures, α_2A_-AR mainly recognizes Gα_i_ (with fewer interactions with G_βγ_ subunits) (Fig. 5a). On α_2A_-AR, the interacting elements include TM3, TM5, TM6, and ICL2 (Fig. 5b). On Gα_i_, the C-terminal α5-helix contributes to the majority of interactions with α_2A_-AR (Fig. 5c). α_2A_-AR is a Gi-coupled receptor, while β_1_-AR couples primarily to Gs and secondarily to Gi^16,21,62,64^. We recently solved the cryo-EM structure of the β_1_-AR and G_i_ complex^21^. The α_2A_-AR–Gi complex and β_1_-AR–Gi complex were compared, revealing that the interactions between the receptors and G_i_ are different (Fig. 5d, e). To functionally support these structural data, receptor-interacting residues on Gi were mutated, and their effects on the signaling initiated by α_2A_-AR and β_1_-AR were examined (Supplementary Fig. 9a–d). Gα-depleted HEK293 cells (in which the genes for all Gα subunits expressed in HEK cells were mutated by using the CRISPR/Cas system) were cotransfected with α_2A_-AR or β_1_-AR and mutated Gα_i1_^49^ (Supplementary Fig. 9). These functional data support the specific interactions revealed by the structures. K345 (located in the C-terminal α5-helix) of Gi interacts with both α_2A_-AR and β_1_-AR (Fig. 5d), and the K345A mutation blocked α_2A_-AR- and β_1_-AR-initiated Gi-mediated cAMP inhibition (Supplementary Fig. 9a, b). K345, as well as nearby I344, L348, and F354 on the α5-helix, seem to form interactions with Gi-coupled GPCRs in general (within packing distance with TM5 and TM6); Ala mutations in these residues individually reduce Gi interactions with the receptors^65,66^. In the α_2A_-AR–Gi complex, K345 of Gi is within the packing distance of R241^5.68^ of α_2A_-AR (Fig. 5d). In the β_1_-AR–Gi complex, K345 of Gi could form interactions with Q237^5.51^ of β_1_-AR (Fig. 5d). Furthermore, L194 (in the loop between β_2_ and β_3_) and F336 (in the C-terminal α5-helix) of Gα_i_ form hydrophobic interactions with F147^34.51^ in ICL2 of β_1_-AR (Fig. 5e). In α_2A_-AR, the equivalent residue is I154^34.51^ (Fig. 5e). I154 in α_2A_-AR and F147^34.51^ in β_1_-AR have different interactions due to side chain differences (Fig. 5e). Compared to I154^34.51^, α_2A_-AR, F147^34.51^ in β_1_-AR seems to insert deeper to form more contacts with F336 (Fig. 5e), and the I154^34.51^ in α_2A_-AR mainly contacts L194 (Fig. 5e). Functional studies revealed that the F336A mutation diminished β_1_-AR-initiated Gi-mediated cAMP inhibition but had a smaller effect on α_2A_-AR-initiated Gi-mediated cAMP inhibition (Supplementary Fig. 9d). On the other hand, L194A impaired α_2A_-AR-initiated and Gi-mediated cAMP inhibition but had a smaller effect on β_1_-AR-initiated and Gi-mediated cAMP inhibition (Supplementary Fig. 9c). Overall, Gi forms fewer interactions with β_1_-AR than with α_2A_-AR. Together, the structural and functional data demonstrate that α_2A_-AR (a primarily Gi-coupled GPCR) and β_1_-AR (a GPCR secondarily coupled to Gi) interact with and signal through Gi differently.

### Mechanism of Gi activation by α_2A_-AR

**Fig. 6:**
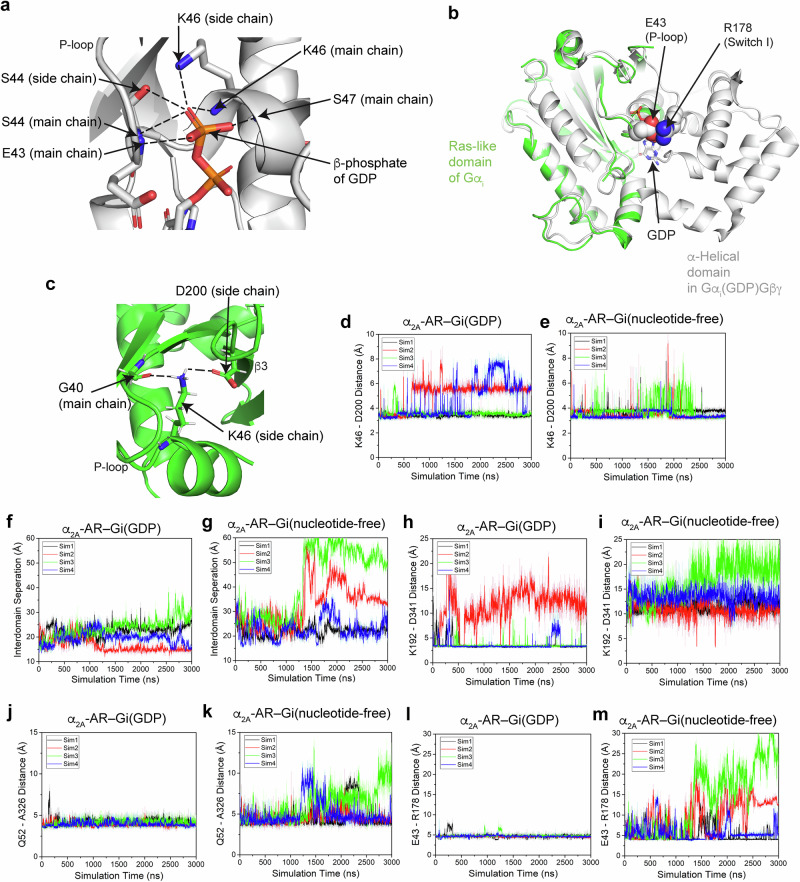
GaMD simulations of G_i_ activation by dexmedetomidine-bound α_2A_-AR. **a** Diagram showing the GDP-binding pocket of Gα_i_. Ionic and hydrogen bonds are depicted as dashed black lines. **b** E43 interacts with R178 in the inactive GDP-bound Gα_i_. **c** K46 interacts with D200 in nucleotide-free Gα_i_. Ionic and hydrogen bonds are depicted as dashed black lines. **d**, **e** The distance between K46 and D200 of Gα_i_ calculated from the GaMD simulations when Gi is bound to GDP (**d**) or is nucleotide free (**e**). **f**, **g** The distance between the Ras-like domain and the α-helical domain of Gα_i_ calculated from the GaMD simulations when Gi is bound to GDP (**f**) or is nucleotide free (**g**). (**h**, **i**) The distance between K192 and D341 of Gα_i_ calculated from the GaMD simulations when Gi is bound to GDP (**h**) or is nucleotide free (**i**). **j**, **k** The distance between Q52 and A326 of Gα_i_ calculated from the GaMD simulations when Gi is bound to GDP (**j**) or is nucleotide free (**k**). **l**, **m** The distance between E43 and R178 of Gα_i_ calculated from the GaMD simulations when Gi is bound with GDP (**l**) or is nucleotide-free (**m**). Four independent 3000 ns GaMD simulations are shown for each condition.

To understand the α_2A_-AR-catalyzed activation of Gi, we compared the GDP/GTP-binding pocket in Gα_i_ in the complex with α_2A_-AR and in the inactive, GDP-bound Gi heterotrimer (Gα_i1_(G203A)Gβ_1_Gγ_2_ trimer, PDB: 1GG2)^67^ (Fig. 6 and Supplementary Figs. 10, 11). Our structure reveals that during activation, the α-helical domain opens by ~77^o^, which is the principal change. This change results in an ~38 Å displacement of its mass center relative to the Ras-like GTPase domain, which anchors the heterotrimer to α_2A_-AR (Supplementary Fig. 10b). Critically, the maximum rotation is limited by the position of Gβγ, with Gβγ acting as a buttress to prevent further rotation (Supplementary Fig. 10c–e).

In complex with α_2A_-AR, Gα_i_ is nucleotide free and represents an intermediate state in the GDP/GTP exchange reaction (Supplementary Fig. 11a). In the inactive state of Gα_i_, the β-phosphate of GDP is coordinated by the sidechains of K46 and S44 in the P-loop and by the main chains of E43, S44, K46 and S47 in the P-loop (Fig. 6a). These structural comparisons revealed that the Switch I region (Linker 2) of Gα_i_ moves away from the GDP/GTP-binding pocket as the α-helical domain rotates away from the Ras-like domain of Gα_i_ (Supplementary Fig. 10). The ionic interaction between E43 on the P-loop and R178 on Switch I was disrupted (Fig. 6b). This E43-R178 interaction might serve as a gate to prevent GDP release (Fig. 6b). In the α_2A_-AR–Gi complex structure, the side chain of K46 interacts with the side chain of D200 on β3 of the Switch II region and the main chain of G40 (Fig. 6c). Moreover, the interaction network between the N-terminal region of Gα_i1_ and C-terminal region of Gα_i1_ in Gα_i1_Gβ_1_Gγ_2_ was disrupted in the α_2A_-AR–Gi complex (Supplementary Fig. 11c). In the Gi trimer, the sidechain of D341 in the α5-helix forms an ionic interaction with the sidechain of K192 in the β2-β3 loop (Supplementary Fig. 11c). In the α_2A_-AR–Gi complex, the rotation and translation of the α5-helix move D341 away and disrupt this interaction (Supplementary Fig. 11c). Furthermore, the sidechain of Q52 in the α1-helix forms a hydrogen bond with the backbone carbonyl of A326 in the β6-α5 loop (the TCAT motif) and interacts with the sidechain of T329 in the α5-helix in the Gi trimer (Supplementary Fig. 11f). In the α_2A_-AR–Gi complex, this contact network is disrupted, leading to movements of the α1-helix, the P-loop and the TCAT motif (Supplementary Fig. 11f). Since these regions form the GDP binding pocket, the combined disruption of these interactions should lead to GDP release and Gi activation.

To functionally test the roles of these identified interacting residues, mutations of these residues were generated. These mutated and wild-type G_i_ constructs were then expressed in Gα-depleted (by CRISPR) HEK293 cells together with α_2A_–AR^49^ (Supplementary Fig. 9e). Forskolin was used to increase cellular cAMP levels, and subsequent epinephrine-induced activation of α_2A_–AR reduced cAMP levels (Supplementary Fig. 9e). These mutations decreased the activation and thus signaling of α_2A_-AR to inhibit cAMP (Supplementary Fig. 9e).

We performed GaMD simulations to examine the G protein activation process^26^ (Fig. 6d, e). We started by examining the complex of dexmedetomidine–α_2A_-AR–Gi and replaced nucleotide-free Gi with inactive GDP-bound Gi, mimicking the starting point of the G protein activation reaction pathway. However, no GDP release was observed from this complex during the 3000 ns GaMD simulations, reflecting the slow kinetics of GDP dissociation (Supplementary Fig. 11b). We then carried out further GaMD simulations by directly removing GDP from the Gi protein. In the absence of GDP, K46 and D200 formed an ionic interaction with a distance of ~3.5 Å between their charge centers during most of the simulation time period (Fig. 6e). In contrast, when GDP was bound to Gi, the salt bridge between K46 and D200 fluctuated between ~3.5 Å and ~6 Å (Fig. 6d). The conformation with an ~6 Å distance revealed that K46 and the β-phosphate of GDP interact (Fig. 6a). Therefore, the GaMD simulations showed that D200 forms a stable salt bridge with K46 in the P-loop in an intermediate step during G protein activation. In contrast, the β-phosphate of GDP competes with this Lys in the inactive state of G proteins.

Additionally, we investigated the conformational changes propagating from the GPCR contact site (the C-terminal α5-helix of Gi that interacts with α_2A_-AR) to the GDP/GTP binding pocket of Gi. In our GaMD simulations, the α-helical domain moved away from the Ras-like domain after ~1400 ns in two of the four independent 3000 ns GaMD simulations, as measured by the distance between the Cα atoms of residues A138 (in the α-helical domain) and E276 (in the Ras-like domain) (Fig. 6g). On the other hand, in the presence of GDP, this interdomain separation was not observed (Fig. 6f). We then inspected the interactions between the α5-helix and structural elements linked to the GDP/GTP-binding pocket and examined the temporal correlation between the interaction changes and α-helical domain separation. One correlated change is that the C-terminal α5-helix adopts a conformation similar to that in the α_2A_-AR–Gi complex (Supplementary Fig. 11e). In this conformation, the α5-helix forms tight interactions with α_2A_-AR. After the second correlated conformational change, the ionic interaction between K192 on the β2-β3 loop and D341 on the α5-helix is disrupted (from ~3 Å in Fig. 6h to ~12.5 Å in Fig. 6i and Supplementary Fig. 11c). After the third correlated conformational change, the interaction between Q52 in the α_1_-helix and A326 in the β6-α5 loop at the end of the α5-helix is disturbed (Fig. 6j,k, Supplementary Fig. 11f). The α_1_-helix is immediately downstream of the P-loop and is directly involved in GDP binding (Fig. 6a). Therefore, both the changes in the K192 and D341 interaction, as well as the Q52 and A326 interaction, propagate the tight binding of the C-terminal α5-helix to α_2A_-AR across ~35 Å to the P-loop (the GDP/GTP-binding pocket). Concomitantly, the ionic interaction between E43 on the P-loop and R178 on Switch I is disrupted, leading to α-helical domain separation (Fig. 6b, l, m). Hence, α_2A_-AR interacts strongly with the C-terminal half of the α5-helix, leading to the disruption of interactions between K192 on the β2-β3 loop and D341 on the α5-helix, as well as between Q52 on the α1-helix and A326 on the β6-α5 loop. These structural changes lead to modification of the GDP/GTP-binding pocket.

### The epinephrine-bound complex is more stable than the dexmedetomidine-bound complex

**Fig. 7:**
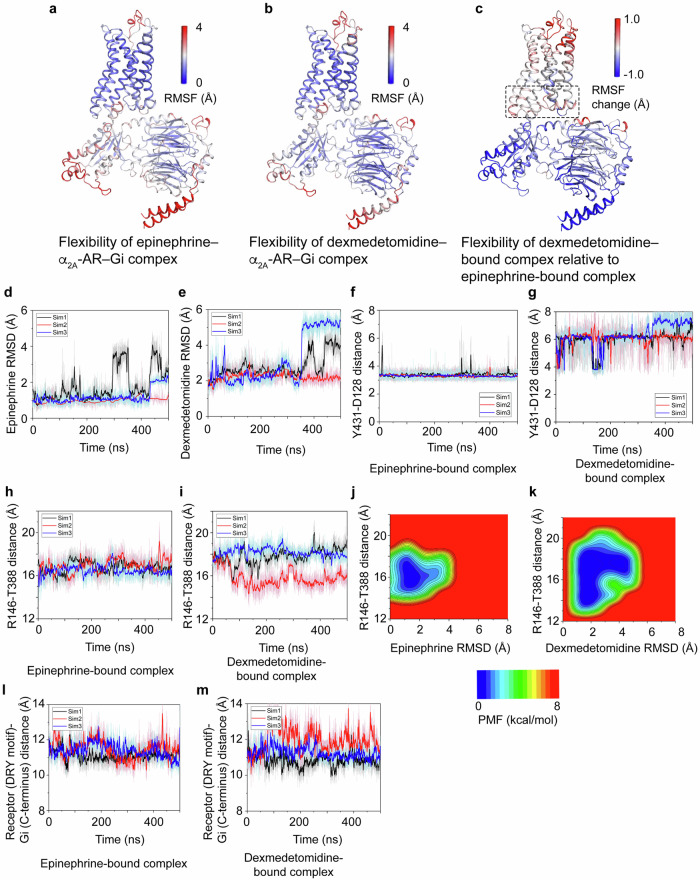
GaMD simulations of the stability of the ligand–α_2A_-AR–Gi complexes. **a**–**c** GaMD simulations showing the changes in the flexibility of the ligand–α_2A_-AR–Gi complexes. The root-mean-square fluctuations (RMSFs) of the epinephrine–α_2A_-AR–Gi complex (**a**) and the dexmedetomidine–α_2A_-AR–Gi complex (**b**) are shown. **c** Changes in the RMSF of α_2A_-AR and Gi in the presence of the dexmedetomidine–α_2A_-AR–Gi complex compared with the presence of the epinephrine–α_2A_-AR–Gi complex. The dashed box indicates the α_2A_-AR–Gi-interacting regions. **d**, **e** Comparison of the agonist flexibilities of the two complexes. The time courses of the root-mean-square deviation (RMSD) of the two agonists in the complexes are shown. **f**, **g** Stable hydrogen bond formed between Y431^7.42^ and D128^3.32^ in the epinephrine–α_2A_-AR–Gi complex. The time courses of the distance between residues Y431^7.42^ and D128^3.32^ in the epinephrine–α_2A_-AR–Gi (**f**) and dexmedetomidine-α_2A_-AR–Gi (**g**) complexes are shown. **h**–**k** Complex dynamic and 2D free energy profiles. Time courses of the distance between the intracellular ends of TM3 and TM6 (measured as the distance in Å between R146 and T388) in the epinephrine–α_2A_–AR–Gi (**h**) and dexmedetomidine–α_2A_–AR–Gi (**i**) complexes. 2D free energy profiles of the agonist RMSD relative to cryo-EM conformations (Å) and R146 and T388 distances (Å) calculated from GaMD simulations in the epinephrine–α_2A_–AR–Gi (**j**) and dexmedetomidine–α_2A_–AR–Gi (**k**) complexes are shown. The time courses of the distance between the DRY motif in α_2A_-AR and the last five C-terminal residues in Gi in the epinephrine–α_2A_-AR–Gi complex (**l**) and in the dexmedetomidine–α_2A_-AR–Gi complex (**m**) are shown.

Finally, we analyzed the structural flexibility of different agonist-bound α_2A_-AR–Gi complexes (Fig. 7). In GaMD simulations of the epinephrine–α_2A_-AR–Gi and dexmedetomidine–α_2A_-AR–Gi complexes, the α_2A_-ARs underwent small fluctuations, except for ICL1 and the extracellular loop 2 (ECL2) (Fig. 7a, b). On the other hand, Gi proteins exhibited greater flexibility than α_2A_-ARs, especially in the C-terminal α5-helix, Switch III, and the αN helix in Gα_i_ and the N-termini of G_βγ_ (Fig. 7a, b). Compared with epinephrine, dexmedetomidine increased the fluctuations in the α_2A_-AR and Gi interface and in α_2A_-AR, especially in ECL2 and the orthosteric ligand-binding site (Fig. 7c). Thus, when the compound is bound with dexmedetomidine, the interaction between α_2A_-AR and Gi is less stable. On the other hand, dexmedetomidine binding to α_2A_-AR led to lower flexibility in the Gi than that observed with epinephrine (Fig. 7c).

Furthermore, in the α_2A_-AR–Gi complexes, the fluctuations were less pronounced in the presence of epinephrine than in the presence of dexmedetomidine (Fig. 7c, d, e). More stable interactions were observed for epinephrine with receptor residues in the epinephrine–α2A-AR–Gi complex. Epinephrine formed a salt bridge with D128^3.32^ and six hydrogen bonds with residues Y431^7.42^, C132^3.36^, S215^5.43^, and S219^5.46^ (Fig. 2). In contrast, dexmedetomidine formed only three hydrogen bonds with the α_2A_-AR residues Y431^7.42^, C132^3.36^, and F427^7.38^ (Fig. 3). Moreover, differences in the residue interactions were observed at the orthosteric ligand-binding site of α_2A_-AR in GaMD simulations of the epinephrine–α2A-AR–Gi and dexmedetomidine–α_2A_-AR–Gi complexes (Fig. 7f–i). In the epinephrine–α2A-AR–Gi complex, the hydrogen bond between residues Y431^7.42^ and D128^3.32^ was stable, while large fluctuations were observed in the dexmedetomidine–α2A-AR–Gi complex (Fig. 7f, g). The ligand RMSD and the TM3-TM6 distance (measured by the distance between R146^3.50^ and T388^6.34^) were used to calculate the 2D free energy profiles of the simulation systems (Fig. 7h–k). The low-energy state of the epinephrine–α2A-AR–Gi complex was sampled with the ligand RMSD and the TM3–TM6 distance centered at (1.2 Å, 15.7 Å) (Fig. 7j), which was consistent with the cryo-EM structure. The dexmedetomidine-α_2A_-AR–Gi complex sampled a larger conformational space and two low-energy wells with a ligand RMSD and a TM3-TM6 distance centered at (1.9 Å, 15.0 Å) and (3.8 Å, 17.2 Å) (Fig. 7k). Additionally, the interaction between α_2A_-AR and Gi is less stable when bound with dexmedetomidine than with epinephrine, as measured by the distance between the DRY motif on TM3 of α_2A_-AR and the last 5 C-terminal residues of Gi (Fig. 7l and m). Together, these data suggest that the dexmedetomidine–α_2A_-AR–Gi complex is less stable than the epinephrine–α_2A_-AR–Gi complex and provide the structural basis for ligand efficacy.

## Discussion

Here, we investigated the mechanisms underlying the molecular recognition of epinephrine by ARs. Surprisingly, we found that epinephrine adopts two different conformations when bound to α_2A_-AR and β-ARs. These two different epinephrine conformations are isomers that result from the rotation around the chemical bond that links the catechol ring and β-carbon of epinephrine. Furthermore, when bound to α_2A_-AR, the β-carbon hydroxyl group of epinephrine faces the extracellular side, and the N-methyl group points to TM7. When bound to β_1_-AR, the β-carbon hydroxyl group faces the intracellular side, and the N-methyl group points toward TM3. Recently, we observed that epinephrine exhibits the same conformation in complex with α_1A_-AR and α_2A_-AR^13^. This new information can be utilized to perform structural modifications and produce α-AR and β-AR selective agonists.

Previous functional studies have extensively elucidated that the ligand-binding residues identified within our structures, namely, D128^3.32^, V129^3.33^, C132^3.36^, T133^3.37^, S215^5.43^, S219^5.46^, W402^6.48^, F405^6.51^, F406^6.52^, Y409^6.55^, F427^7.38^, and Y431^7.42^, play pivotal roles in orchestrating the intricate interactions between α_2A_-AR and its ligands (Fig. 2a, and Supplementary Figs. 6, 12)^53,54,60,62,68^. Although epinephrine and dexmedetomidine occupy a common binding site, their engagement with α_2A_-AR diverges significantly. Through dedicated functional analyses, we investigated select ligand-binding residues (namely, D128^3.32^, T133^3.37^, S215^5.43^, S219^5.46^, F405^6.51^, F406^6.52^, Y409^6.55^, and Y431^7.42^) to determine their distinctive responses to epinephrine and dexmedetomidine. While different ligands engage similar residues within the orthosteric ligand-binding pockets of ARs, the nuances of these interactions subtly deviate among the ligands. The roles of the corresponding residues in accommodating distinct ligands within ARs are remarkably diverse. Moreover, recent advancements in structure-based docking and optimization have unveiled novel α_2A_-AR agonists, which serve as nonopioid analgesics and are characterized by attenuated side effects^69^. Compared to canonical agonists such as dexmedetomidine and norepinephrine, these innovative agonists, which are endowed with novel chemotypes, forge more potent interactions with F427^7.3869^. These findings underscore the pivotal significance of F427^7.38^ in conferring subtype-specific discernment of ligands.

Our data also revealed that α_2A_-AR (a primarily Gi-coupled GPCR) and β_1_-AR (a GPCR primarily coupled to Gs and secondarily coupled to Gi) interact differently with Gi. These results contribute to our knowledge on the coupling specificity among GPCRs and G proteins, which is not fully understood. Previously, we solved and compared the cryo-EM structures of β_1_-AR with Gs or with Gi and revealed that β_1_-AR recognizes Gs versus Gi through three-dimensional interactions^21,27^. The overall β_1_-AR–G-protein complex structure (not the α5-helix structure alone) dictates β_1_-AR–G-protein interaction modes, and the different modes of interaction between β_1_-AR–Gs and β_1_-AR–Gi contribute to the differences in activation selectivity and efficiency^21^. Furthermore, we conducted a structural comparison between our α_2A_-AR–Gi complex and an α_2A_-AR–Go complex (Supplementary Fig. 13). Upon superimposing the α_2A_-ARs, we observed variations in the orientations of Gi and Go (Supplementary Fig. 13a). Notably, the most prominent distinction was observed in the N-termini position within the G-proteins (Supplementary Fig. 13a). This difference could be attributed to the incorporation of scFv16 in the α_2A_-Go complex. Additionally, we identified disparities in the interactions between α_2A_-AR and G proteins. For instance, K345 on Gi was closely packed against R241^5.68^ of α_2A_-AR (Supplementary Fig. 13b, c), while in Go, the corresponding residue was A345 (Supplementary Fig. 13b, d). Notably, A345 of Go did not interact with any residues on α_2A_-AR (Supplementary Fig. 13b, d). These findings collectively highlight the distinct interactions between α_2A_-AR and Gi versus Go.

In summary, we investigated the interactions between epinephrine and the α and β families of ARs and revealed new information on the structural characteristics of the interactions. The data show that epinephrine adopts different conformations to interact with these families of ARs. We also provide structural information on the binding of epinephrine to a GPCR-G protein complex. We further elucidated the selectivity of GPCR-G-protein coupling by investigating the coupling modes of Gi to α_2A_-AR and β_1_-AR. GaMD simulations were used to propose a possible sequential order for the activation of Gi by α_2A_-AR. These findings advance our knowledge of the mechanisms underlying GPCR and G protein activation.

## Supplementary information

Supplementary Information

## Data Availability

The cryo-EM reconstructions of the epinephrine–α_2A_-AR–Gi complex and the Dexmedetomidine–α_2A_-AR–Gi complex have been deposited in the Election Microscopy Data Bank (EMDB) under ID codes EMD-45425 and EMD-45426, respectively. The corresponding atomic models have been deposited in the Protein Data Bank (PDB) under ID codes 9CBL and 9CBM, respectively.
