# Supplementary material for: Distinct binding conformations of epinephrine with α- and β-adrenergic receptors
Source: Exp Mol Med. 2024 Sep 2;56(9):1952–66. doi: 10.1038/s12276-024-01296-x (PMC11447022; doi:10.1038/s12276-024-01296-x)
Supplement: Supplementary file 1 — Supplementary Information [file 12276_2024_1296_MOESM1_ESM.pdf]

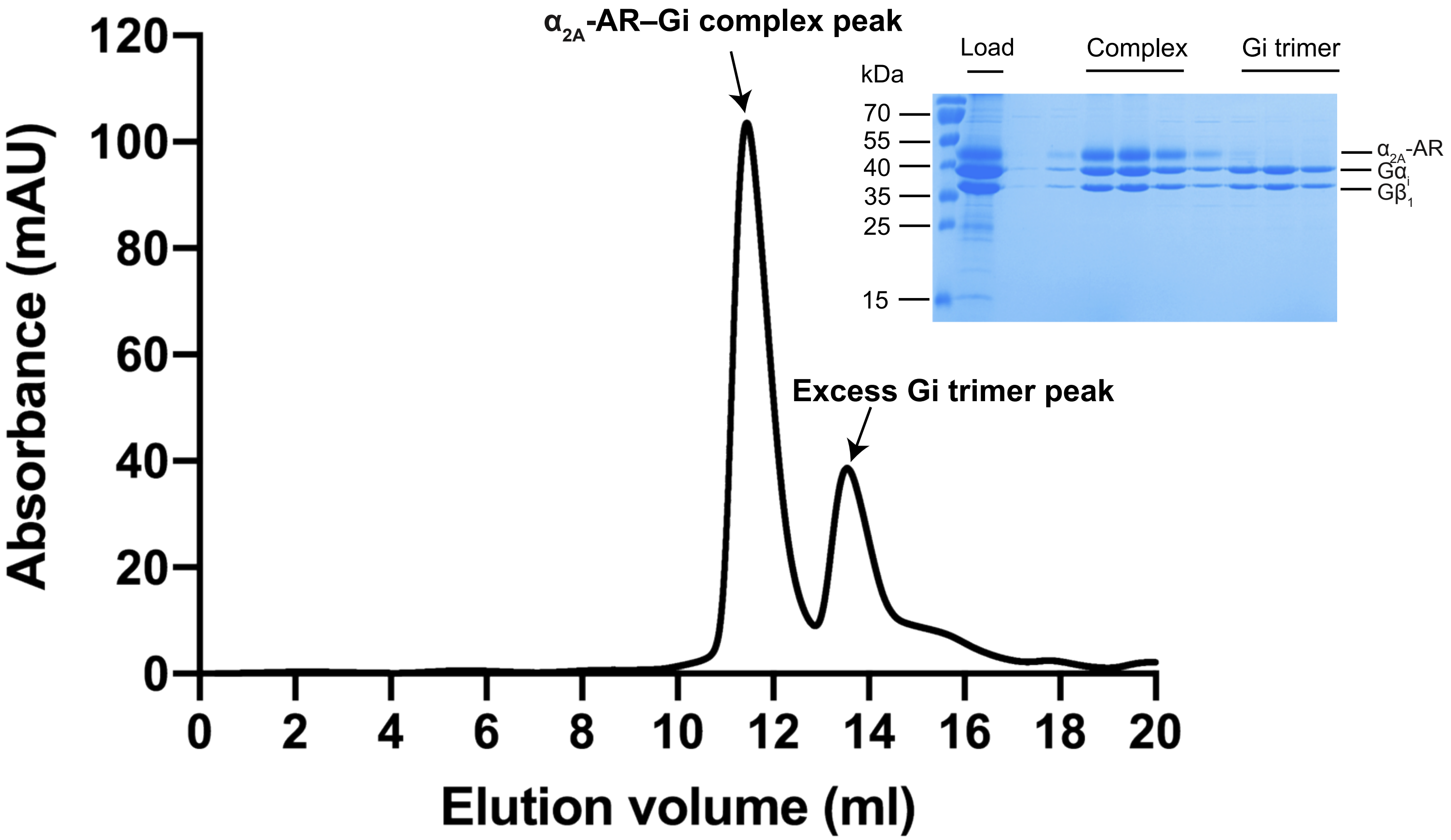

Supplementary Fig. 1. **Purification of the α_{2A}-AR–Gi complex.**
Size-exclusion chromatography profile and corresponding SDS-PAGE gel of the purified epinephrine–α_{2A}-AR–Gi complex.

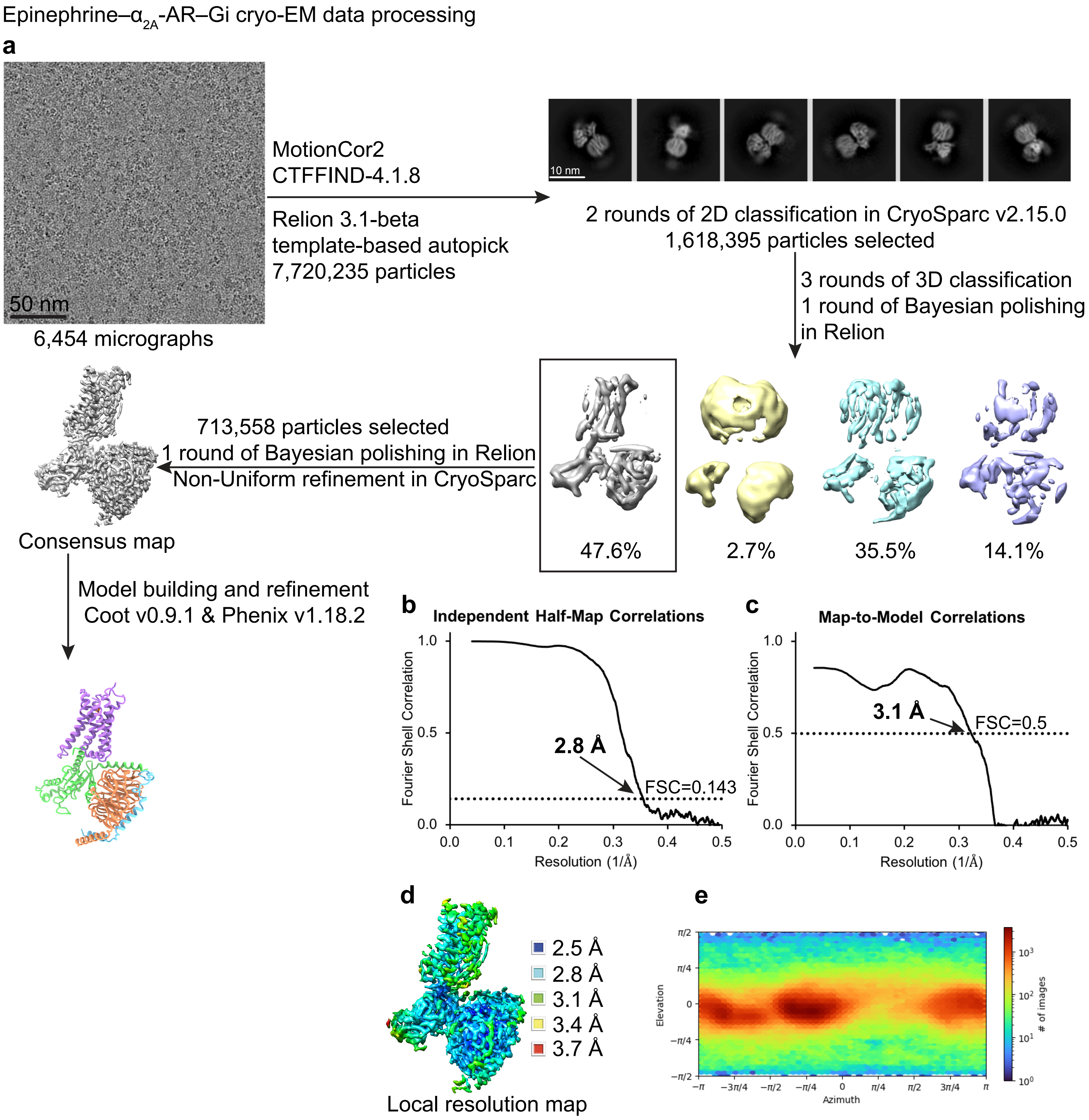

Supplementary Fig. 2. Epinephrine–α_{2A}-AR–Gi cryo-EM data processing.

a, Cryo-EM data processing workflow of epinephrine–α_{2A}-AR–Gi complex. **b**, Fourier shell correction curves of consensus map. **c**, Cross-validation of consensus map to epinephrine–α_{2A}-AR–Gi complex model.
d, Local resolution of consensus map. **e**, Angular distribution calculated in cryoSPARC.

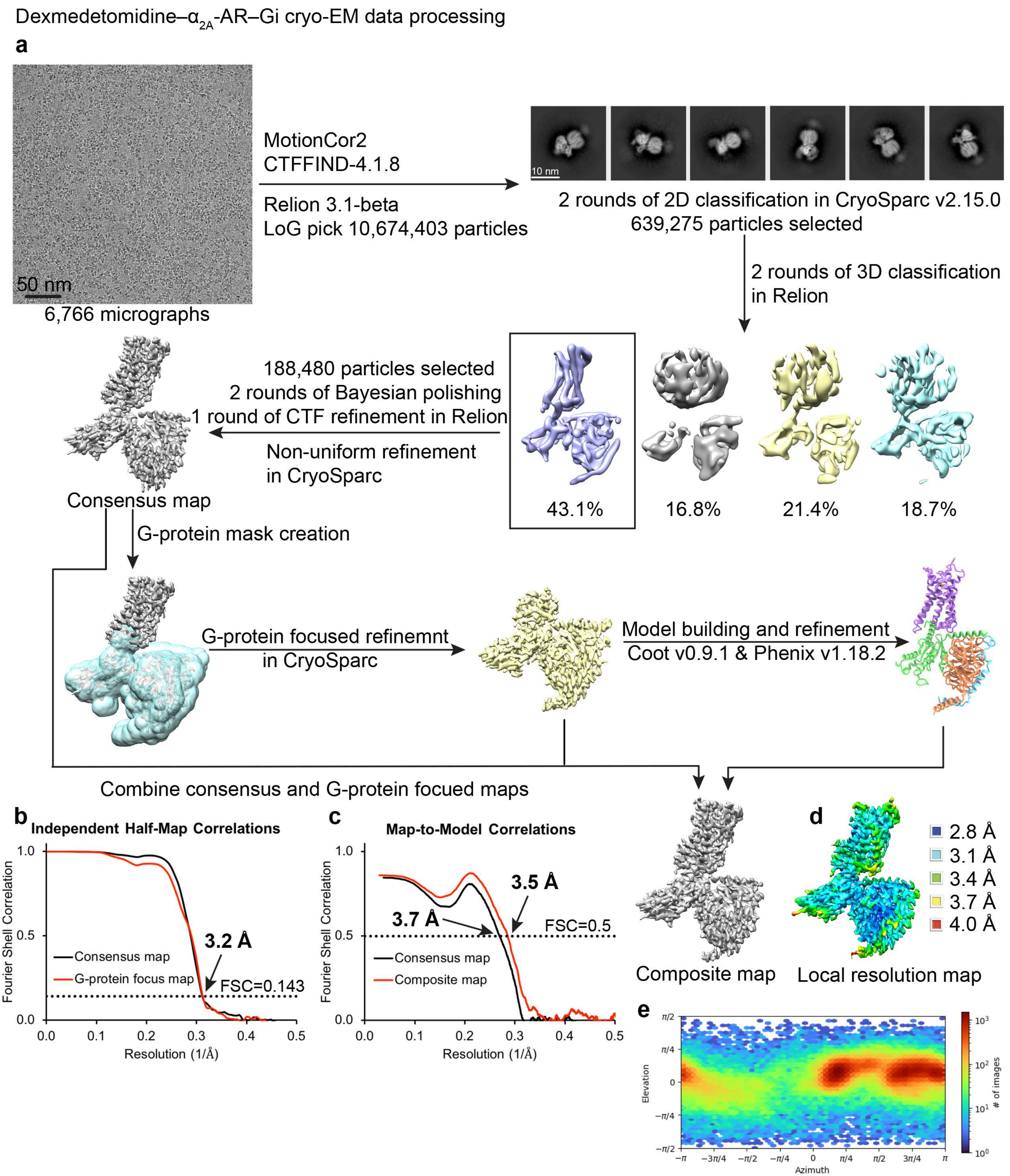

Supplementary Fig. 3. Dexmedetomidine–α$_{2A}$-AR–Gi cryo-EM data processing.
a, Cryo-EM data processing workflow of dexmedetomidine–α$_{2A}$-AR–Gi complex. **b**, Fourier shell correction curves of both consensus map and G-protein focus map. **c**, Cross-validation of consensus and composite maps to dexmedetomidine–α$_{2A}$-AR–Gi complex model. **d**, Local resolution of consensus map. **e**, Angular distribution calculated in cryoSPARC.

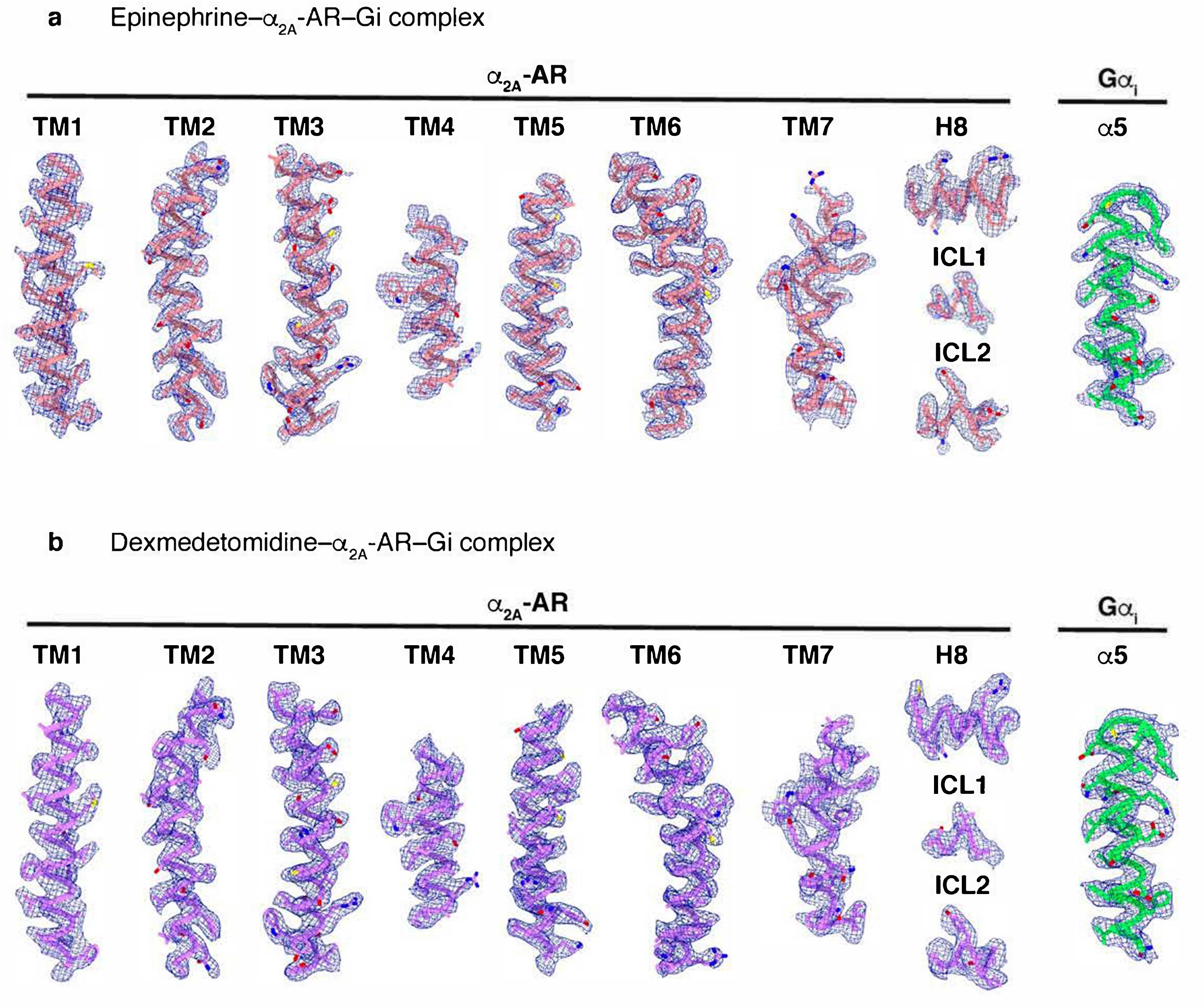

Supplementary Fig. 4. Cryo-EM maps versus refined structures. Cryo-EM density maps and models are shown for all seven transmembrane α-helices, helix 8, and intracellular loops 1 and 2 of of α_{2A}-AR, and α5 helix of Gα_i, for epinephrine–α_{2A}-AR–Gi complex **(a)** and dexmedetomidine–α_{2A}-AR–Gi complex **(b)**.

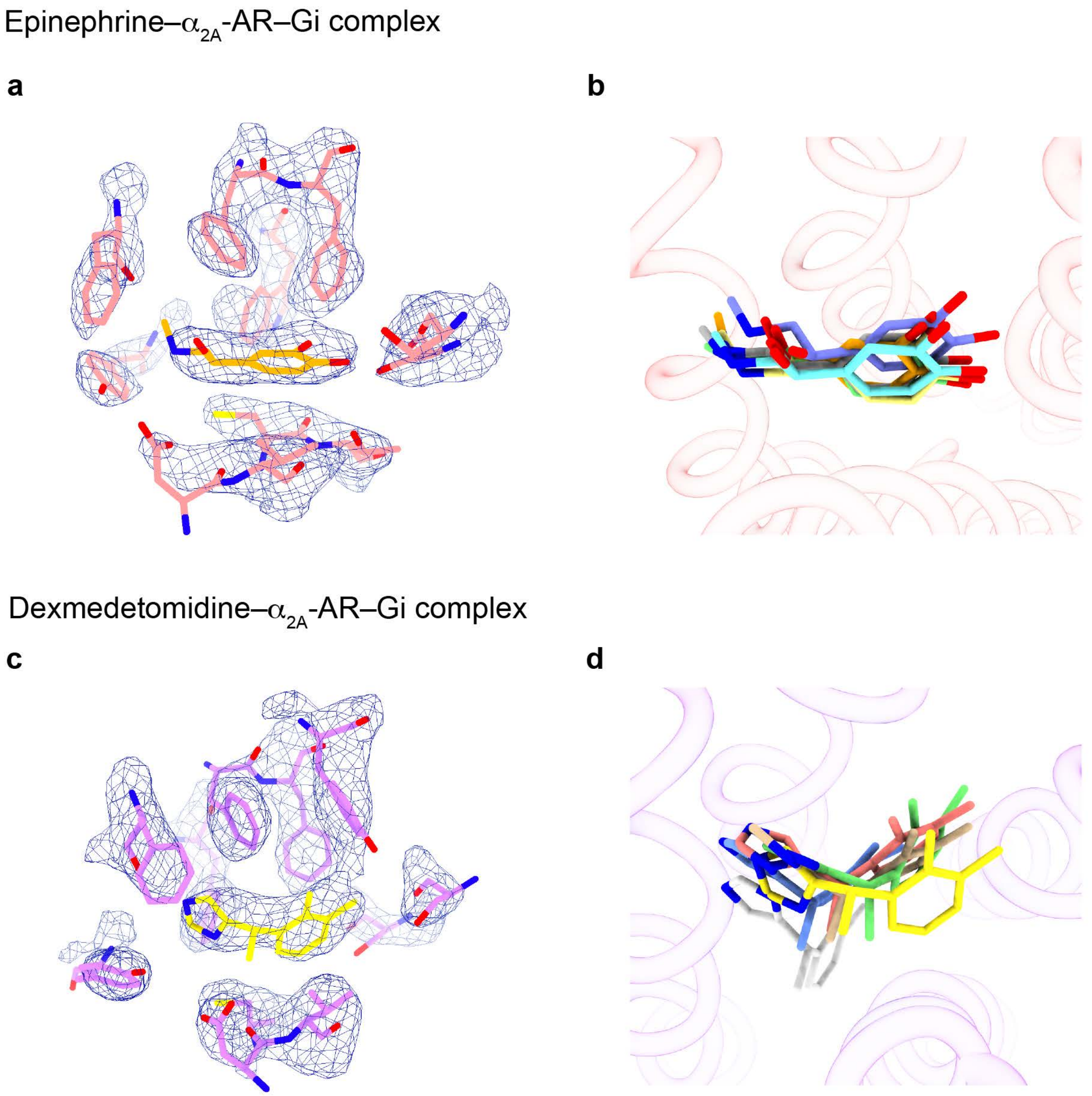

Supplementary Fig. 5. Cryo-EM structures and MD simulations of bound ligands. a, c, Cryo-EM density maps of bound epinephrine and dexmedetomidine and surrounding residues in the complexes of epinephrine–α_{2A}-AR–Gi and dexmedetomidine–α_{2A}-AR–Gi. **b, d,** Five top clusters of each ligands from MD simulations are shown in comparison with cryo-EM poses for epinephrine (orange) and dexmedetomidine (yellow). The ranking of the top 5 poses is based on their populations in the MD trajectories. For epinephrine, cluster 1: 91.4%; cluster 2: 5.2%; cluster 3: 1.9%; cluster 4: 1.1%; cluster 5: 0.2%. For dexmedetomidine, cluster 1: 58.7%; cluster 2: 11.5%; cluster 3: 11.4%; cluster 4: 8.2%; cluster 5: 5.5%.

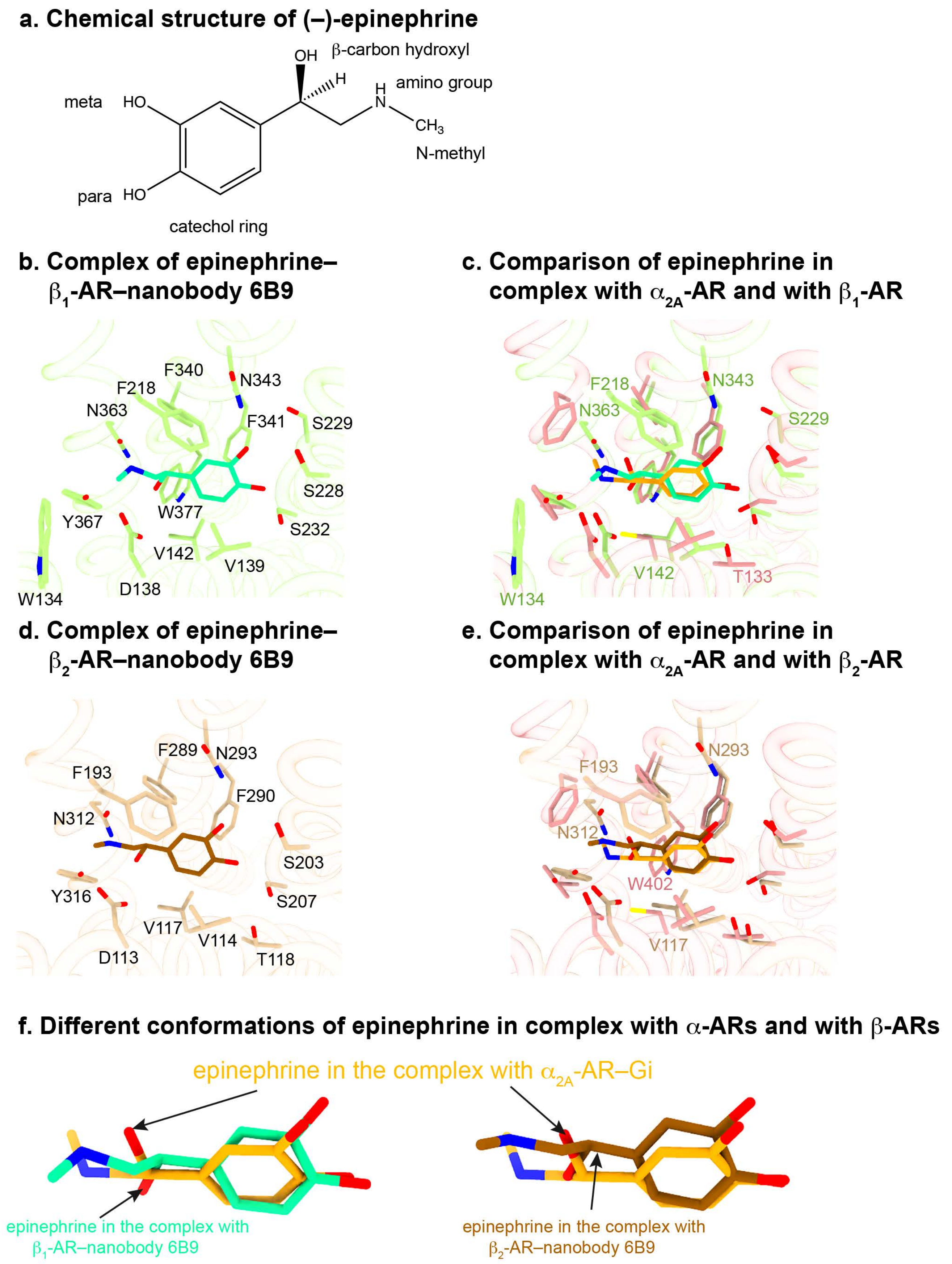

Supplementary Fig. 6. **Different conformations of epinephrine. a,** Chemical structure of epinephrine. **b,** epinephrine in the complex of epinephrine–β_1-AR–nanobody 6B9. **c,** Comparison of epinephrine in complex with α_{2A}-AR and with β_1-AR. **d**, Epinephrine in the complex of epinephrine–β_2-AR–nanobody 6B9. **e**, Comparison of epinephrine in complex with α_{2A}-ARand with β_2-AR. **f**, Conformational flexibility of epinephrine.

a. Chemical structure of dexmedetomidine

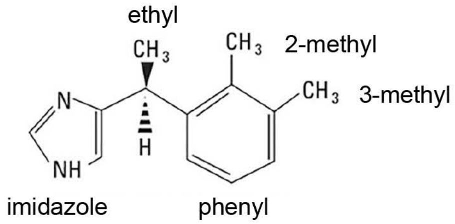

b. Complex of dexmedetomidine–α_{2B}-AR–Gi

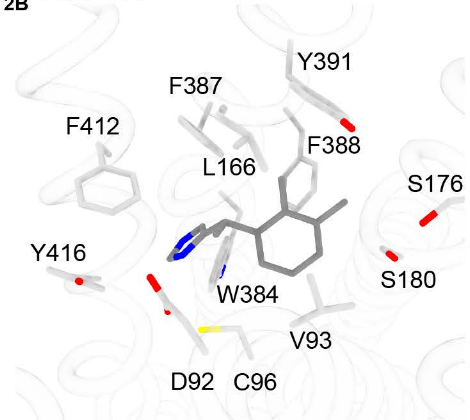

c. Comparison of dexmedetomidine in complex with α_{2A}-AR–Gi and with α_{2B}-AR–Gi

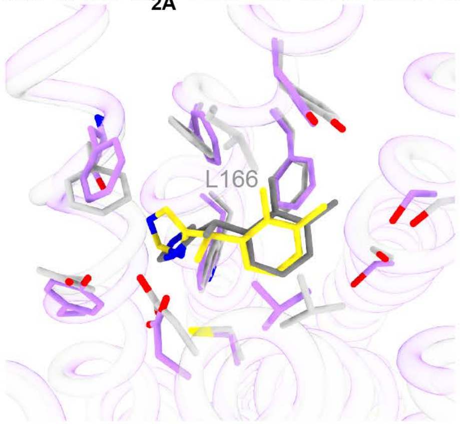

d. Complex of dexmedetomidine–α_{2A}-AR–Go

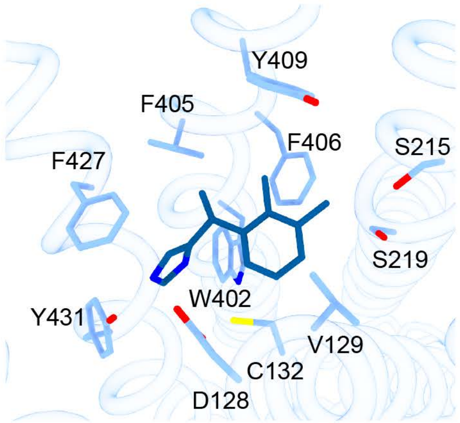

e. Comparison of dexmedetomidine in complex with α_{2A}-AR–Gi and with α_{2A}-AR–Go

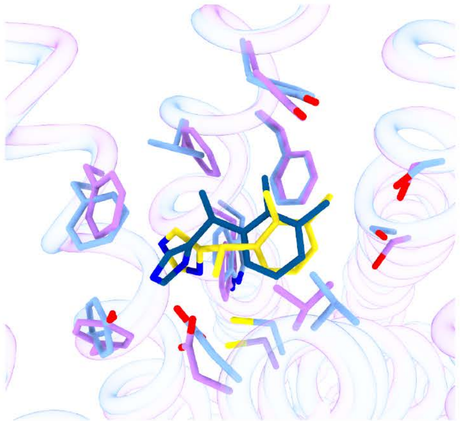

f. Conformational flexibility of dexmedetomidine

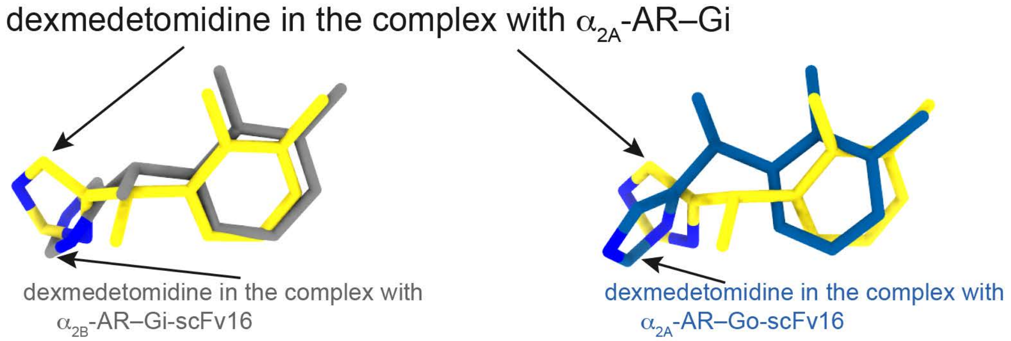

Supplementary Fig. 7. **Flexible configurations of dexmedetomidine. a,** Chemical structure of dexmedetomidine. **b,** Dexmedetomidine in the complex of dexmedetomidine–α_{2B}-AR–Gi. **c,** Comparison of dexmedetomidine in complex with α_{2A}-AR–Gi and with α_{2B}-AR–Gi. **d**, Dexmedetomidine in the complex of dexmedetomidine–α_{2A}-AR–Go. **e**, Comparison of dexmedetomidine in complex with α_{2A}-AR–Gi and with α_{2A}-AR–Go. **f**, Conformational flexibility of dexmedetomidine.

α_{2A}-AR constructs	Epinephrine Emax (%)	*p* values	EC_{50} (nM)	*p* values	Receptor expression (% of WT)	Dexmedetomidine Emax (%)	*p* values	EC_{50} (nM)	*p* values	Receptor expression (% of WT)
WT	38.3±2.9		4.6±0.2		100	30.1±4.2		3.2±1.6		100
D128A	97.8±4.4	<0.0001	43.1±17.6	0.0193	103	64.8±4.8	0.0007	65.5±31.2	0.0260	105
T133A	93.8±3.7	<0.0001	117.9±17.3	0.0003	104	59.2±3.9	0.0009	92±18.1	0.0011	96
S215A	86.7±2.1	<0.0001	36.1±17.2	0.0338	104	39.7±4.1	0.0472	15.4±1.5	0.0006	106
S219A	78.6±2.8	<0.0001	4.6±1.9	1.000	95	52.8±3.6	0.0021	8.3±1.1	0.0104	98
F405A	72.4±5.2	0.0006	1.3±0.5	0.0004	94	46.3±2.4	0.0044	20.5±2.6	0.0006	99
F406A	65.0±2.4	0.0002	1.8±0.4	0.0004	95	51.1±3.5	0.0027	7.1±1.9	0.0530	103
Y409A	94.4±2.7	<0.0001	16.2±5.8	0.0256	101	39.7±1.2	0.0190	12.4±8.6	0.1426	97
Y431A	119.0±2.1	<0.0001	ND		105	70.6±2.7	0.0001	84.7±39.1	0.0226	95

Supplementary Fig. 8. **Functional studies of agonist-interacting residues of α_{2A}-AR.** The data presented in Figs. 2h and 3i are analyzed here. Data are shown as mean ± SD of three independent experiments. ND: could not be determined. α_{2A}-AR plasmids were transfected into CHO cells that were then used for the cAMP assays.

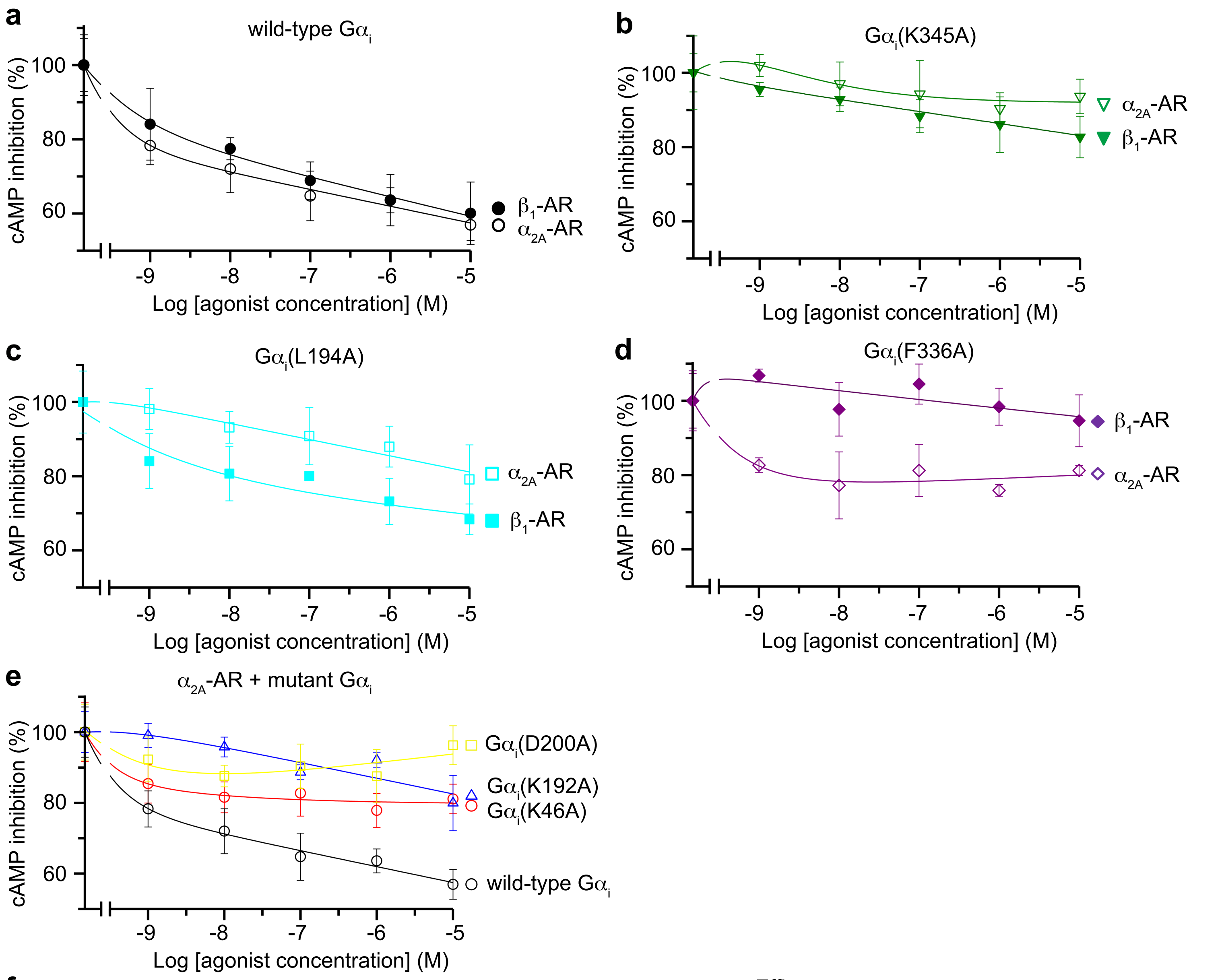

f

Receptors	Constructs	cAMP levels (pmol/mg protein) (without agonist)	cAMP levels (pmol/mg protein) (with 10 μM agonist)	Efficacy (cAMP inhibition, % of without agonist)	p values	Receptor expression (% of WT)	Gi protein expression (% of WT)
α_{2A}-AR	WT	164.1±11.6	93.4±6.9	56.9±4.2		100	100
	K46A	178.8±14.8	145.0±7.5	81.1±4.2	0.0021	108	99
	K192A	172.2±10.0	137.6±13.5	79.9±7.8	0.0108	95	100
	L194A	179.2±2.2	141.8±16.7	79.1±9.3	0.0196	96	97
	D200A	168.2±13.3	162.0±9.3	96.3±5.5	0.0006	100	96
	F336A	226.0±16.6	183.5±3.1	81.2±1.4	0.0007	97	95
	K345A	132.5±6.8	124.0±6.1	93.6±4.6	0.0005	93	105
β_1-AR	WT	103.9±8.5	62.3±8.8	60.0±8.4		100	100
	K46A	108.7±9.9	81.5±5.2	75.0±4.8	0.0549	ND	ND
	K192A	129.4±8.6	84.4±10.1	65.2±7.8	0.4760	ND	ND
	L194A	155.9±13.0	106.6±6.4	68.4±4.1	0.1946	102	96
	D200A	116.3±9.9	119.6±9.6	102.8±8.2	0.0032	ND	ND
	F336A	114.1±9.2	107.9±8.0	94.6±7.0	0.0054	97	101
	K345A	123.4±12.3	102.0±6.9	82.7 ±5.6	0.0176	95	97

Supplementary Fig. 9. Functional studies of Gi activation by α_{2A}-AR and β_1-AR. a-d, Effects of wild-type and mutant $G\alpha_i$ on the cAMP inhibition signaling initiated by α_{2A}-AR or β_1-AR. Some of the responses did not reach saturation; it might be due to the relative expression levels of the receptor and the G-protein. **e,** Effects of wild-type and mutant $G\alpha_i$ on the cAMP inhibition signaling initiated by α_{2A}-AR. The curves for wild-type $G\alpha_i$ + α_{2A}-AR in **a** and **e** are the same. **f**, Analysis of the data presented in **a-e**. Data are shown as mean ± SD of three independent experiments. ND: not determined. $G\alpha_{i1}$ plasmids were transfected into Gα-depleted HEK293 cells that were then used for the cAMP assays.

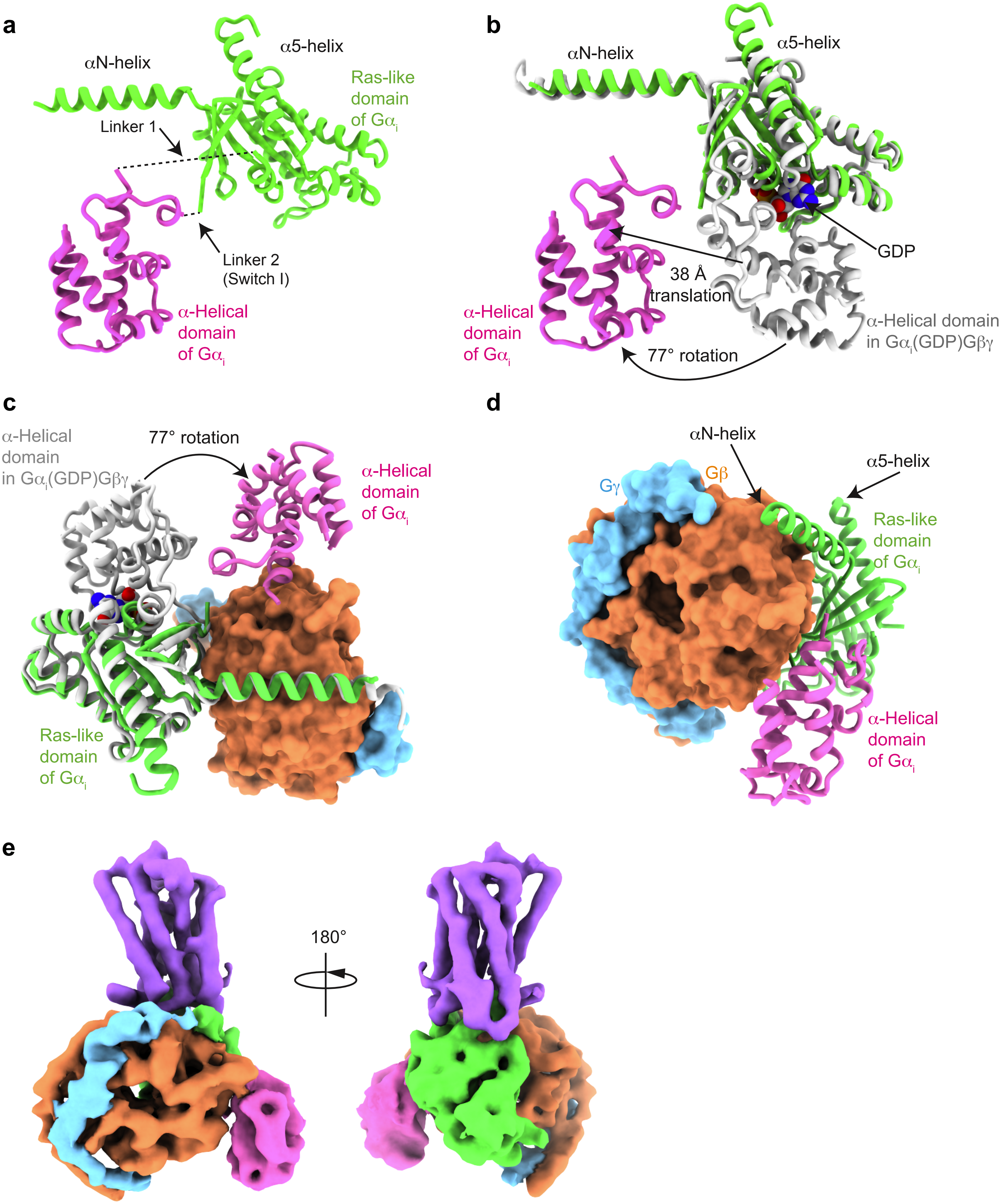

Supplementary Fig. 10. **Rotational opening of the α-helical domain during G-protein activation by GPCRs**. **a**, Structure of Gα$_i$ in the complex of α$_{2A}$-AR–Gi shows the rotational opening of the α-helical domain away from theRas-like domain. **b**, Comparison of the structures of Gα$_i$ in the complex of α$_{2A}$-AR–Gi (in green and magenta) and in the inactive GDP-bound Gi crystal structure (in gray; PDB: 1GG2). **c**, View from the receptor towards the cytoplasmic end shows the rotation of the α-helical domain from the position in inactive Gi (in gray) to the location in the α$_{2A}$-AR–Gi complex (in magenta). **d**, View from Gβγ towards the Ras-like domain shows the position of the α-helical domain relative to Gβ. **e,** Cryo-EM density map of the dexmedetomidine-α$_{2A}$-AR–Gi complex low-pass filtered to 6 Å.

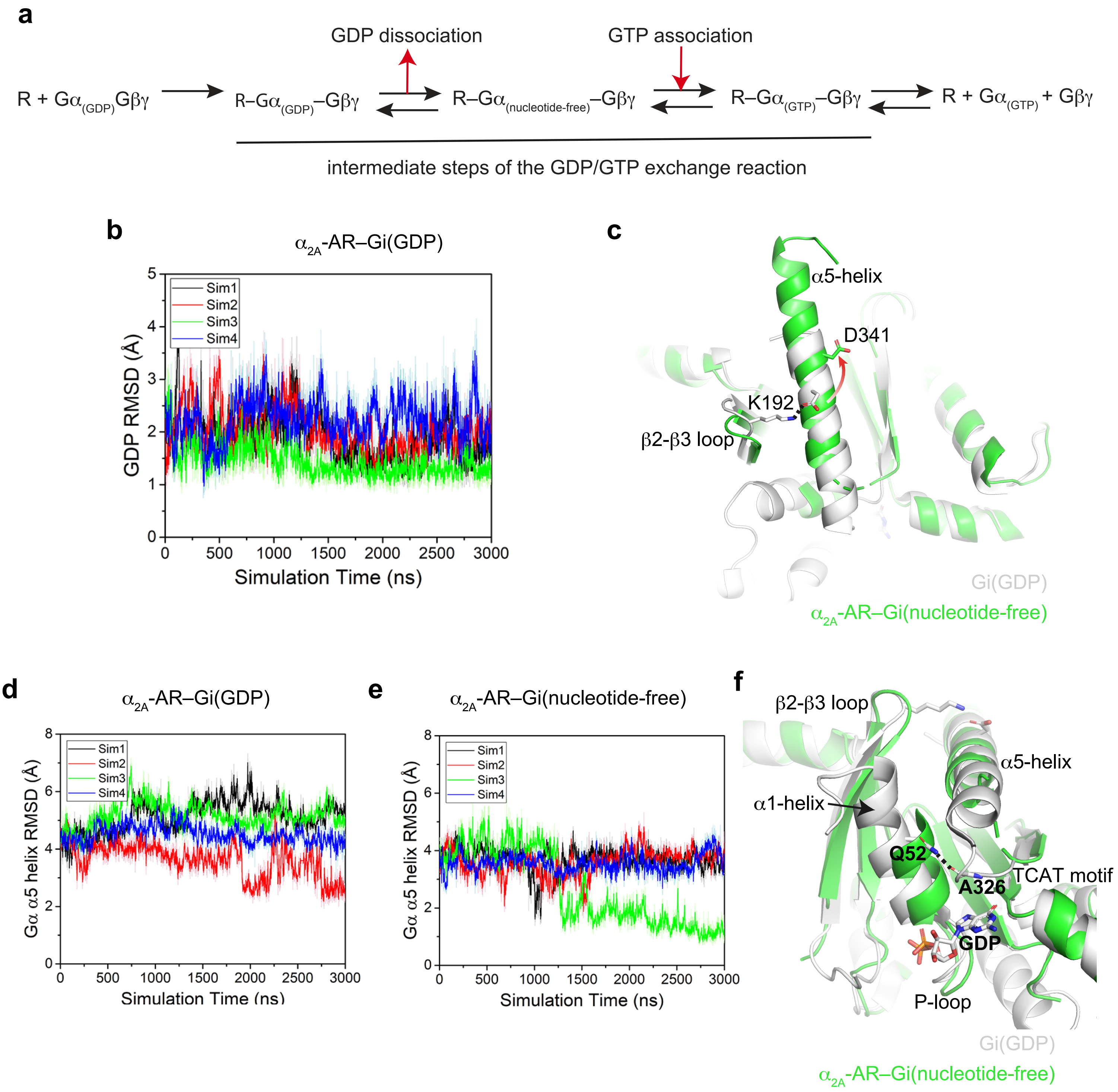

Supplementary Fig. 11. **GaMD simulations of Gi activation by α_{2A}-AR.** **a**, A schematic representation of a GPCR- catalyzed GDP/GTP exchange on a G-protein. **b**, The GDP RMSD from the GaMD simulations when Gi was bound with GDP. **c**, Disruptions of intra-molecular interactions of $G\alpha_i$ during Gi activation. An ionic interaction between the sidechain of D341 in the α5-helix and the sidechain of K192 in the β2-β3 loop in the inactive $G\alpha_i$(GDP)Gβγ trimer (in gray) is broken. **d**,**e**, The C-terminal α5-helix RMSD relative to the α5-helix in the cryo-EM structure of α_{2A}-AR–Gi during the GaMD simulations when Gi was bound with GDP (**d**) or nucleotide-free (**e**). **f**, An ionic interaction between the sidechain of Q52 and the mainchain of A326 in the inactive $G\alpha_i$(GDP)Gβγ trimer (in gray) is broken. Four independent 3000 ns GaMD simulations are shown for each condition.

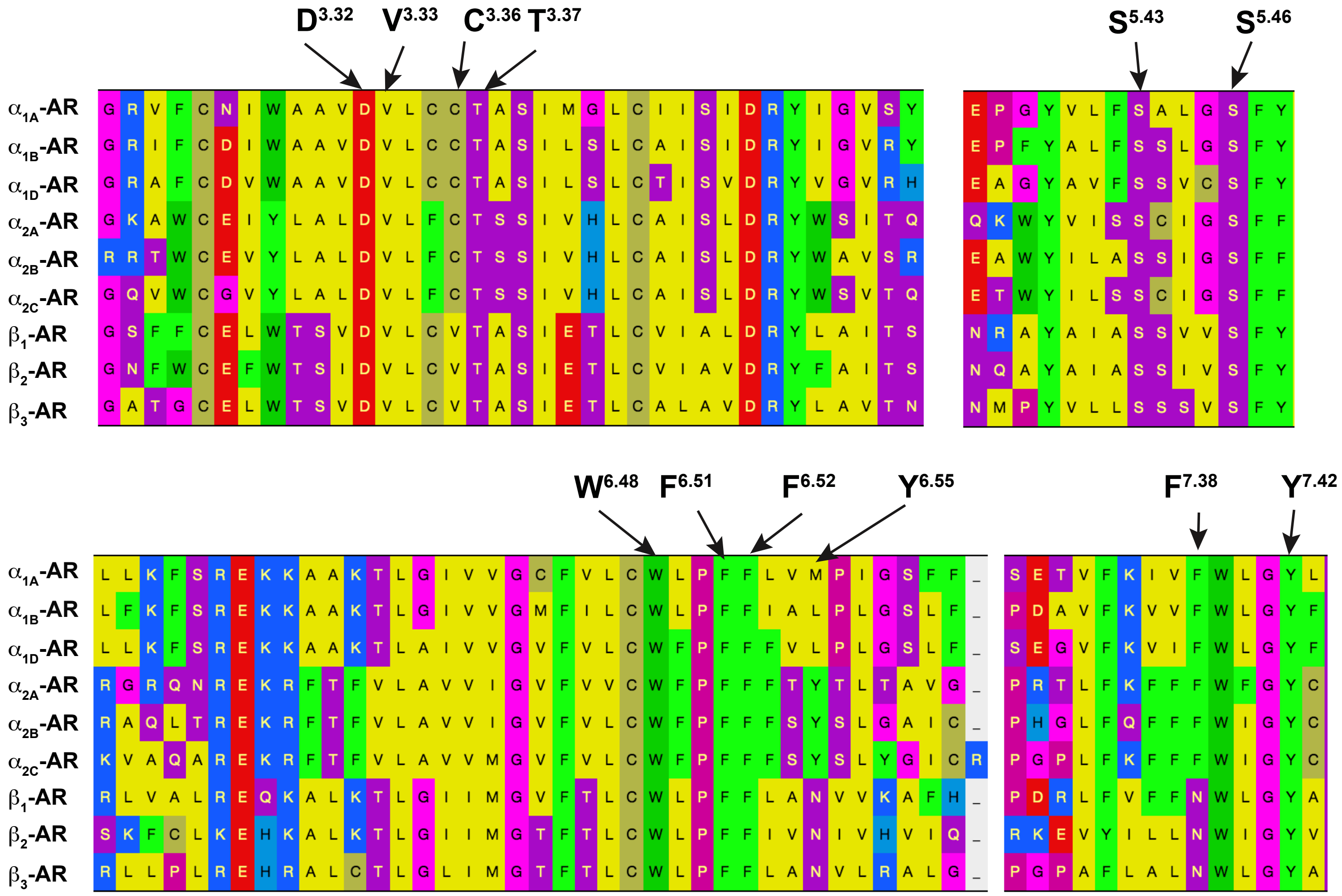

Supplementary Fig. 12. Structure-based sequence alignment of all human ARs generated in GPCRdb. Ligand interacting residues are labeled.

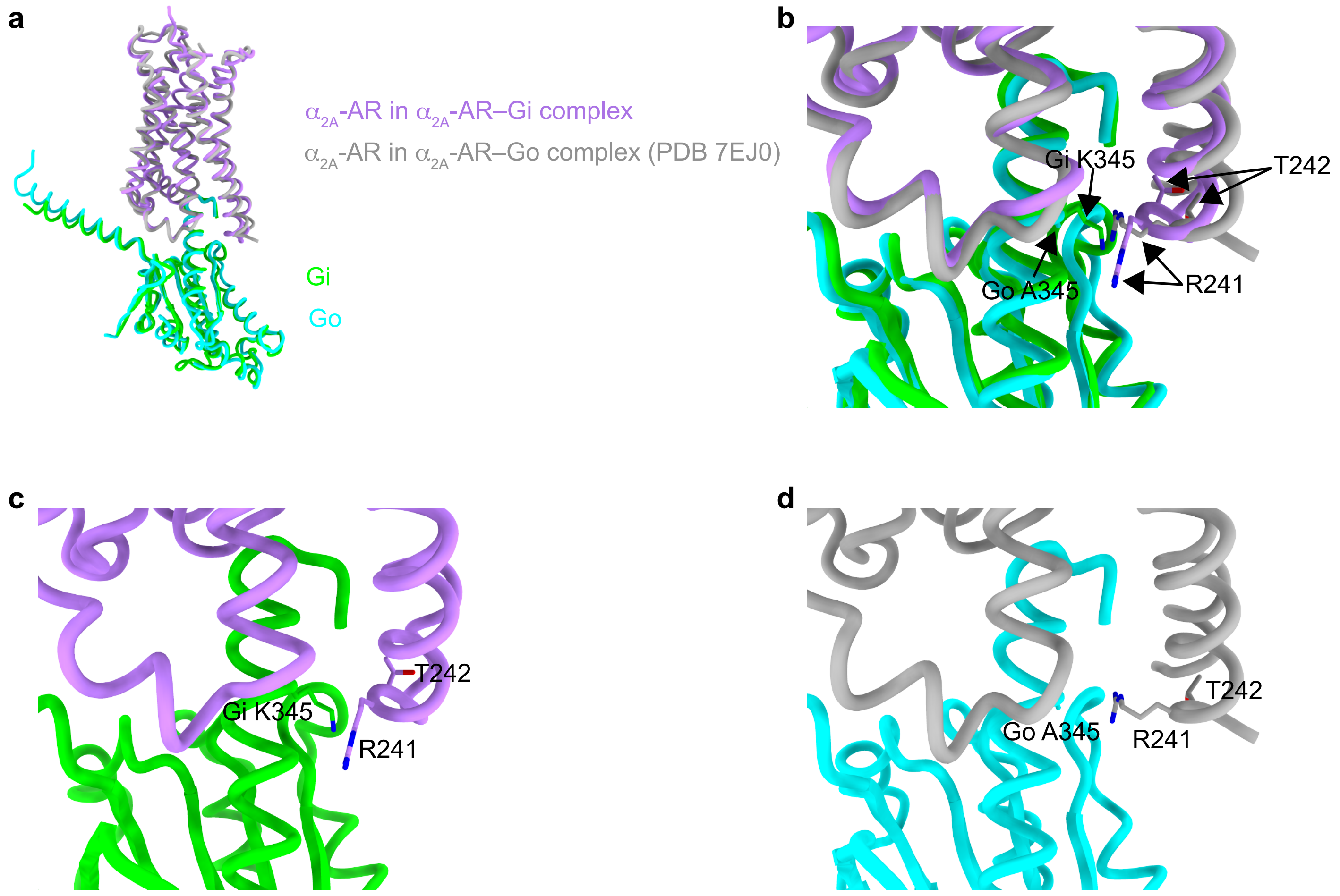

Supplementary Fig.13. **Comparison of α_{2A}-AR–Gi and α_{2A}-AR–Go.** **a**, Structural comparison of the dexmedetomidine–α_{2A}-AR–Gi complex and the dexmedetomidine–α_{2A}-AR–Go complex (in the presence of scFv16) (PDB 7EJ0). **b-d**, K345 on Gi is packed against R241 of α_{2A}-AR (**b** and **c**). A345 of Go does not interact with any residues on α_{2A}-AR (**b** and **d**).

Supplementary Table 1. Cryo-EM data acquisition, reconstruction and model refinement statistics

	**Epinephrine–α_{2A}-AR–Gi**	**Dexmedetomidine–α_{2A}-AR–Gi**
**Cryo-EM acquisition and processing**		
EMDB accession #	EMD-45425	EMD-45426
Magnification	81,000x	81,000x
Voltage (kV)	300	300
Total electron exposure (e^- /Å^2)	50	51
Exposure time (s)	4	2
Defocus range (µm)	-0.8 to -1.8	-0.8 to -2
Pixel size (Å)	1.07	1.082
Final particles (no.)	713,558	188,480
Resolution (masked, Å)	2.8	3.2
**Model Refinement**		
PDB ID	9CBL	9CBM
Model resolution (Å)	2.8/3.1	3.1/3.5
FSC threshold	0.143/0.5	0.143/0.5
RMS deviations		
Bond length (Å)	0.005	0.005
Bond angle (˚)	0.670	1.008
Ramachandran plot		
Favored (%)	95	91
Allowed (%)	5	9
Disallowed (%)	0	0
Rotamer Outliers (%)	0	0
Validation		
MolProbity score	1.55	1.90
Clashscore	4.32	6.86
